# Searching for Natural Aurora a Kinase Inhibitors from Peppers Using Molecular Docking and Molecular Dynamics

**DOI:** 10.3390/ph16111539

**Published:** 2023-10-31

**Authors:** Paweł Siudem, Łukasz Szeleszczuk, Katarzyna Paradowska

**Affiliations:** Department of Organic and Physical Chemistry, Faculty of Pharmacy, Medical University of Warsaw, Banacha 1, 02-093 Warsaw, Poland; lukasz.szeleszczuk@wum.edu.pl (Ł.S.); katarzyna.paradowska@wum.edu.pl (K.P.)

**Keywords:** Aurora A, TRPV1, MCF-7, capsaicin, peppers, molecular docking

## Abstract

Natural products are the precursors of many medicinal substances. Peppers (*Piper*, *Capsicum, Pimienta*) are a rich source of compounds with potential multidirectional biological activity. One of the studied directions is antitumor activity. Little research has been carried out so far on the ability of the compounds contained in peppers to inhibit the activity of Aurora A kinase, the overexpression of which is characteristic of cancer development. In this study, molecular docking methods, as well as molecular dynamics, were used, looking for compounds that could inhibit the activity of Aurora A kinase and trying to determine whether there is a relationship between the stimulation of the TRPV1 receptor and the inhibition of Aurora A kinase. We compared our results with anticancer activity studied earlier on MCF-7 cell lines (breast cancer cells). Our research indicates that the compounds contained in peppers can inhibit Aurora A. Further in vitro research is planned to confirm the obtained results.

## 1. Introduction

The Aurora kinase family, including aurora kinases A, B, and C, is a group of highly conserved serine/threonine kinases that are important for the correct transition through mitosis [1]. Errors in mitosis can lead to genomic changes that are typically associated with tumorigenesis [2]. Although the faithful process of mitosis is connected with the activity of Aurora kinases, its overexpression may be connected with the development and course of some cancers, such as laryngeal, ovarian, breast, colorectal and gastric cancer [3]. This is associated with the partial degradation of the p53 protein, the physiological function of which leads to damage in the apoptotic pathway. Aurora kinase, due to the phosphorylation of p53 (Ser315) protein, inhibits this activity. Moreover, as described earlier, the overexpression of Aurora kinases induces chemoresistance in breast cancer cells [4]. The overexpression of Aurora A may result in resistance to the treatment of breast cancer with paclitaxel via the transactivation of the FOXM1 promoter [5]. Since the therapeutic process requires the inhibition of overexpressed Aurora kinase, the use of several small molecule aurora kinase inhibitors as potential anticancer therapeutic agents has recently been investigated. Although there are numerous studies of synthetic molecules [6,7,8], natural products or directly derived compounds play a crucial role in the discovery of new drugs.

One of many well-known and studied active molecules derived from natural products is capsaicin from chili peppers (*Capsicum*). The traditional use of peppers and their use in modern societies has raised interest in their biological applications, including cytotoxic and antiproliferative effects. Hui-Chung et al. reported that Aurora A protein increased in response to cisplatin and was degraded upon combined treatment with capsaicin with cisplatin, suggesting that the Aurora A-mediated signaling pathway is responsible for the resistance to cisplatin in cisplatin-resistant gastric cancer cell lines [9]. 

Therefore, the aim of this study was to find out if the other compounds contained in the *Piper*, *Capsicum* and *Pimenta* (the main genera of peppers consumed worldwide), as well as their analogs, can act as Aurora A (Aurka) inhibitors. Since capsaicin is one of the best-known activators of the ion-channel type TRPV1 receptor, we looked for the connection between TRPV1 and Aurora inhibiting activity. 

A set of ligands was used composed of 16 compounds occurring in *Piper*, *Capsicum* and *Pimenta* genera or their derivatives (see Table 1). The compounds were selected according to their reported antitumor activity against breast cancer. Molecular docking calculations were carried out to Aurka and TRPV1.

This is the first step in the study of natural compounds such as Aurka inhibitors. Currently, in silico studies often precede the in vivo and in vitro tests and show the direction in which to conduct further biological research [10]. Molecular docking has become one of the increasingly important tools for drug discovery [11]. This method is less time- and cost-consuming and allows one to choose the best promising compounds for in vitro tests. Molecular docking is widely used in studies of anticancer properties of small molecules. It has been used to describe the disruption of binding between Bcl-2 and Bax proteins involved in the induction of apoptosis [12]. The additional use of molecular dynamics calculations enables the description of the changes in the structural integrity of proteins [13] or to predict the structure of the protein [14]. Our results will be used to plan further molecular dynamics and in vitro studies. 

**Table 1:** Summary of docking results with logP, molar mass, and IC50 (MCF-7) values.

No.	Ligand	Ligand Name	IC50 [µM]	LogP	Molar Mass
1	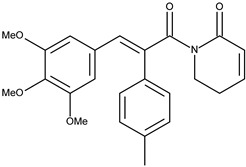	7-(4-methylphenyl)piperlongumine	4.9 [15]	3.36	407.47
2	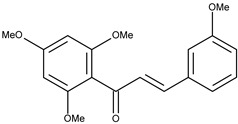	flavokawain B	9.4 [16]	2.67	328.36
3	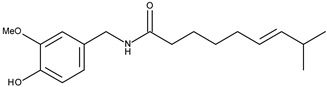	Capsaicin	53 [17]	3.53	305.42
4	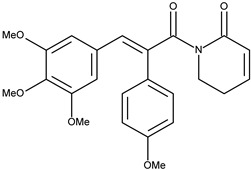	7-(4-methoxyphenyl)piperlongumine	1.6 [15]	2.64	423.47
5	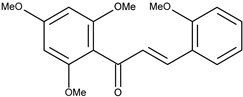	2,2′,4′,6′-tetramethoxychalcone	8.9 [16]	2.67	328.36
6	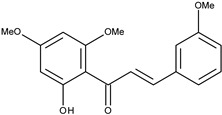	3,2′,4′,6′-tetramethoxychalcone	10.5 [16]	2.63	314.34
7	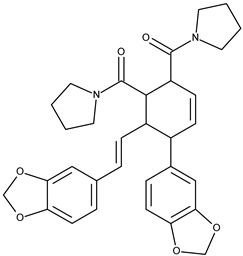	chabamide F	49.9 [18]	3.68	542.63
8	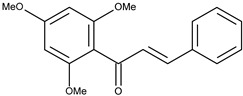	2′,4′,6′-tetramethoxychalcone	9.4 [16]	2.92	298.34
9	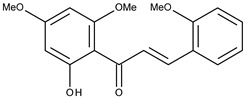	2′-hydroxy-2,4′,6′-trimethoxychalcone	10.3 [16]	2.63	314.34
10	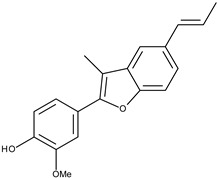	eupomatenoid-5	21.2 [19]	3.33	293.34
11	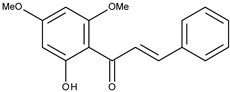	2′-hydroxy-4′,6′-dimethoxychalcone	38.4 [16]	2.89	284.31
12	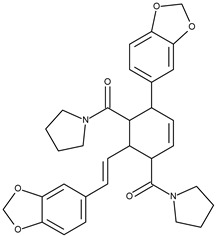	chabamide G	51.4 [18]	3.68	542.63
13	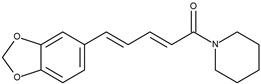	Piperine	99 [20]	2.72	285.34
14	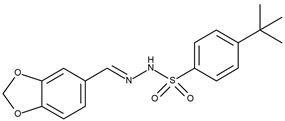	4-tertbutyl-N′-1,3-benzodioxolebenzenesulphonohydrazone	142.4 [21]	4.76	360.43
15	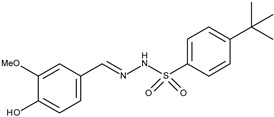	4-tertbutyl-N′-(4-hydroxy-3-methoxy)benzylbenzenesulphonohydrazone	144.6 [21]	4.54	362.44
16	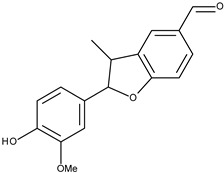	3′-methoxymiliumollin-10-al	169.1 [22]	3.16	284.31

## 2. Results and Discussion

Firstly, the validation of the molecular docking protocol has been performed. A set of 12 ligands with known affinity to Aurka has been created. Kd values showing affinity to Aurora A protein were taken from PubChem (AID 624919). The structures of ligands, Kd values, and results of docking are included in Table S1 (supplementary materials). Docking scoring functions were compared with the literature Kd values. Then, the linear regression was analyzed, and coefficients of determination (R^2^) were calculated. The results are presented in Figure S1 (supplementary materials). The highest R^2^ value has been observed using Surlex (0.67). AutoDock and Glide gave less significant correlations (0.41 and 0.21, respectively). Therefore, we have chosen Surflex as the software that gives the best results. 

In the second step of validation, docking of the Aurka inhibitor present in the 3H10 crystal structure was performed, and its conformation was compared with that of the crystal structure (Figure S2, supplementary materials). Both conformations are similar to each other, which is an additional confirmation of the correctness of the simulations.

Finally, we checked the chosen program (Surflex) on ligands with lower affinity to Aurka (Kd > 10 according to PubChem AID 624919). The results (Figure S3, supplementary materials) show that Total Score values for these ligands are lower than 5.00. It indicates that this model may be used also for compounds with lower affinity to Aurka.

After validation, molecular modeling simulations of a set of 16 ligands (Table 1) to the Aurka protein were performed. In the first stage, docking was carried out using three different programs (Surflex, AutoDock, and Glide). However, the optimization of the program used for docking has been performed earlier, so we wanted to compare results from these three programs. It would be interesting if the comparison of docking scores with biological activity measured as IC50 towards MCF-7 cells and affinity measured as Kd would be comparable. Table 1 shows a list of ligands, the IC50 values determined on MCF-7 cell lines (breast cancer cells), log *p* values, and the molar mass of each ligand.

The results obtained from docking are presented in Table 2 and Table 3.

To better understand the contribution of various energy terms to the binding of ligands to Aurka, the MM/GBSA calculations have been performed (Table 2). It has been found that for the ligands, the binding results mostly from van der Waals interactions and non-polar (lipophilic) contributions to the solvation free energies. Positive values of the polar contributions to the solvation free energies found for all ligands (except **14** and **15**) indicates that the desolvation of the ligand is thermodynamically non-favorable as all those compounds are well soluble in water due to the multiple interactions they form with this solvent, which is a direct consequence of multiple hydrogen bond acceptors present in those molecules.

To compare the results obtained from docking using Sybyl, we performed docking using AutoDock. The results of docking are summarized in Table 3. The docking binding energy, intermolecular energy, total internal energy, torsional energy and unbound energy allowed to distinguish the real binding conformations of ligands on Aurora kinase from energetically unfavorable ones like in the study of Reghav et al. [23].

The results from AutoDock suggest that all ligands have a similar affinity to Aurka. The overall comparison of binding energy values showed the highest binding energy (−8.97 kcal/mol) for ligand 1. Ligands **1** and **10** have calculated IC in the range of nM, which corresponds to the lowest binding energy. Additionally, ligands **15** and **16** have the lowest affinity to Aurka using Surflex and AutoDock. 

The correlation was statistically significant (Pearson correlation, *p* < 0.05). The results obtained from docking are expressed by the Total Score value related to the stability of the ligand–protein complex (dissociation constant). The obtained values make it possible to rank the compounds according to the supposedly increasing affinity for Aurka. 

Then, we compared the Total Score values obtained for the tested ligands and the Total Score values calculated in the same way for Aurka inhibitors with known biological activity. Three Aurka inhibitors were selected: AZD1152, hesperidin and PHA-73935. The results obtained from docking, along with the literature IC50 value (Aurka), are summarized in Table 4.

Total Score values obtained for known Aurka inhibitors are in the range of 5.817–8.043 This means that by comparing only the values of the scaling functions, it can be assumed that ligands 1–8 with Total Score values within this range can have the properties inhibiting the activity of Aurora A kinase. As the analysis of the Total Score function alone is insufficient, the interactions exhibited by the ligands in the Aurka active site should also be analyzed.

To verify the correctness of the molecular docking performed in Surflex, we analyzed the arrangement and interactions of hesperadin in the Aurka active site and then compared them with the results published earlier. Talele and McLaughlin published a study on the molecular docking and molecular dynamics of Aurka inhibitors [28].

According to their study, hesperadin forms hydrogen bonds with Ala 213 (O···HN) and Glu211 (-NH···O) backbone atoms. Moreover, hydrophobic contacts with Thr217 and Leu263 are necessary to stabilize the ligand–protein complex. The sulfonamide group is located next to Lys162 within the hydrogen bonding distance. Talele and McLaughlin confirmed the formation of hydrogen bonds using molecular dynamics simulations [28].

**Figure 1:**
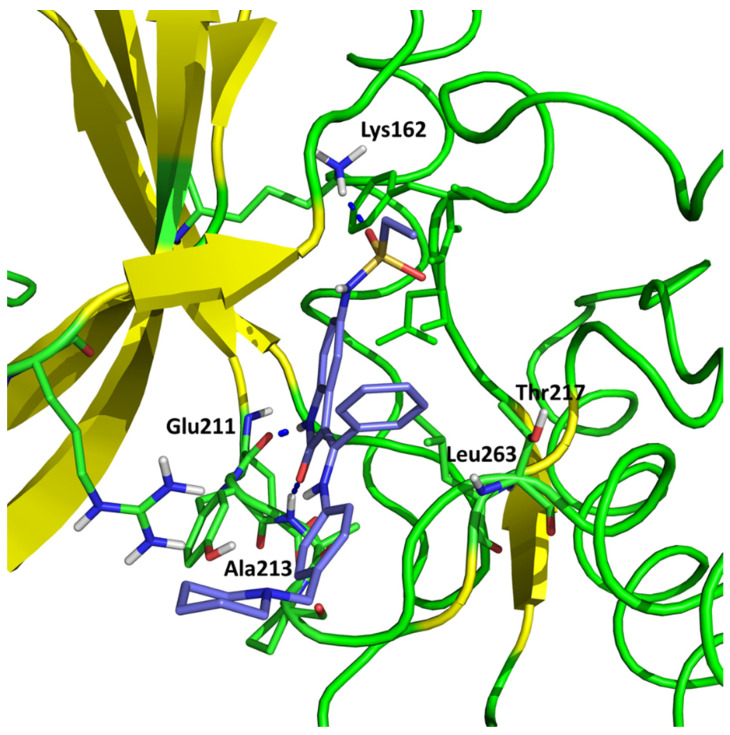
Conformation of hesperadin in the active site of Aurka from molecular docking studies.

Our results are in agreement with the above results. We obtained the conformation of hesperadin stabilized using Ala213 and Lys162 hydrogen bond interaction (2.089 Å and 1.976 Å, respectively) and localized in the active pocket made by the hydrophobic group of Thr217 and Leu263 (Figure 1).

**Figure 2:**
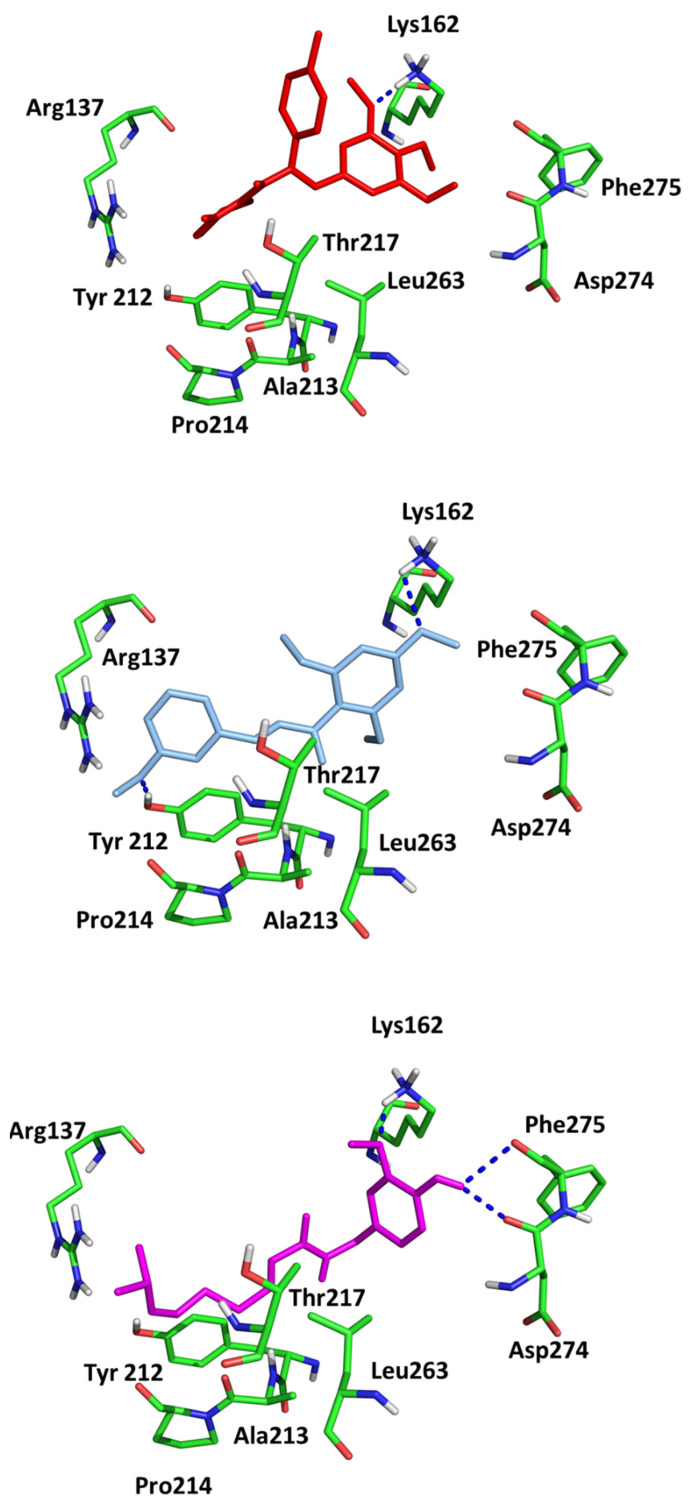
Conformations of (**1**) (red), (**2**) (blue) and (**3**) (magenta) in the active site of Aurka from molecular docking studies.

Since in earlier stages, we found that the results obtained with Surflex correlate best with IC50 and Kd values, the tested compounds have Total Score values similar to known Aurora A kinase inhibitors, and the configuration and interactions of hesperadin obtained in our simulations correspond to those published previously, we also analyzed the interactions in the active site of ligands **1**–**3**, according to their best Totals Score values (Table 1). All three ligands were docked in the pocket formed by Arg137, Lys162, Pro214, Ala213, Tyr212, Asp274, Thr 217, Phe275 and Leu263. It was the same pocket in which hesperadin had been docked. All ligands 1–3 form hydrogen bonds with Lys162 by Me-O···NH interactions (**1**—1.847 Å, **2**—2.625 Å, **3**—1.980 Å). Ligand **2** is also stabilized in the active site by forming a hydrogen bond with Tyr212 (1.906 Å) and ligand 3 with Asp274 (2.346 Å) and Phe 275 2.677 Å). The ligand–protein interactions are presented in Figure 2. 

The results of the in silico study (molecular docking) suggest that a large group of compounds tested by us can potentially act as Aurka inhibitors. The analysis of the docking site shows the importance of the ligand hydrogen bond formation with the amino group of Lys162. This bond is usually formed by oxygen of the -OMe group attached to the aromatic ring.

**Figure 3:**
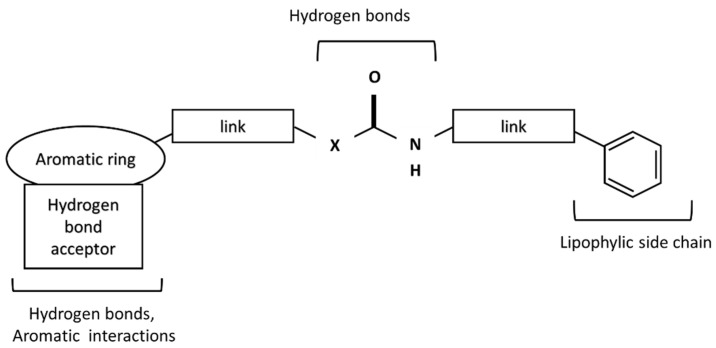
A scheme of TRPV1 receptor ligand.

Surprisingly, this structure is important for TRPV1 ligands. The structures responsible for the formation of hydrogen bonds are present in all TRPV1 ligands. The general scheme of the TRPV1 ligand structure is shown in Figure 3.

In the central part, urea (X = N), amide (X = C), or thiourea moiety may be present. The aromatic group can be monocyclic or bicyclic. The π–π interactions between the ring and the corresponding aromatic fragment in the receptor affect the additional stabilization of the molecule in the active site. Such interactions are observed, for instance, for capsaicin, which can form a specific conformation with the bent side chain in the active site of the receptor [29]. Other capsaicinoids can exist in a similar conformation in the active site of TRPV1 [30]. The lipophilic side chain interacts with amino acids of the hydrophobic side chain present in the TRPV1 binding site. The connectors act as a “scaffold” that links both elements together [31].

Since the same moieties are involved in hydrogen bond formation in the active site of Aurka and TRPV1, we thought that there could be a relationship between Aurora– and TRPV1–ligand interactions. 

**Figure 4:**
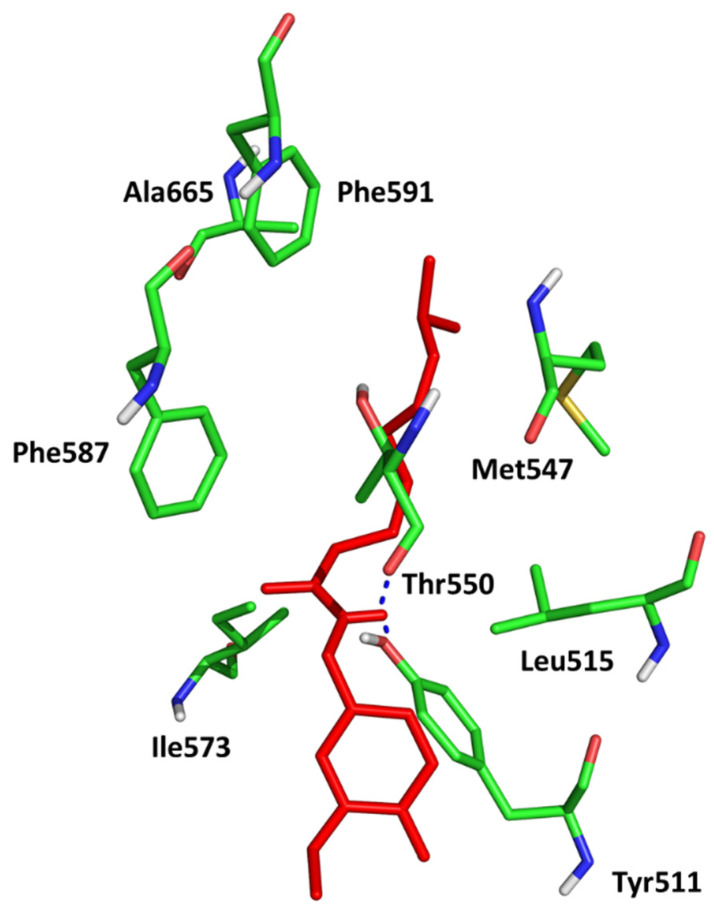
Conformation of capsaicin (red) in the active site of TRPV1 (5IS0).

The results of the molecular docking to TRPV1 receptor expressed as Total Score function are presented in Supplementary Materials in Table S1. As we expected, the best affinity was observed for capsaicin. The conformation of capsaicin was similar to those described by Darre and Domene [32]. The interactions include hydrogen bonding to Thr550 and Tyr511 and stabilization in the hydrophobic cluster formed by Leu515, Ile573, Phe587 (aromatic ring) and Met547, Ala665, Phe591 (aliphatic chain; Figure 4).

To observe the relationship between the measured variables, PCA (Principal Component Analysis) was applied. Data include not only Total Score functions from molecular docking to Aurka and TRPV1 (only Surflex was included because it showed the best correlation with IC50 values for MCF-7) but also logP, molar mass and IC50 (MCF-7 cells) values. 

**Figure 5:**
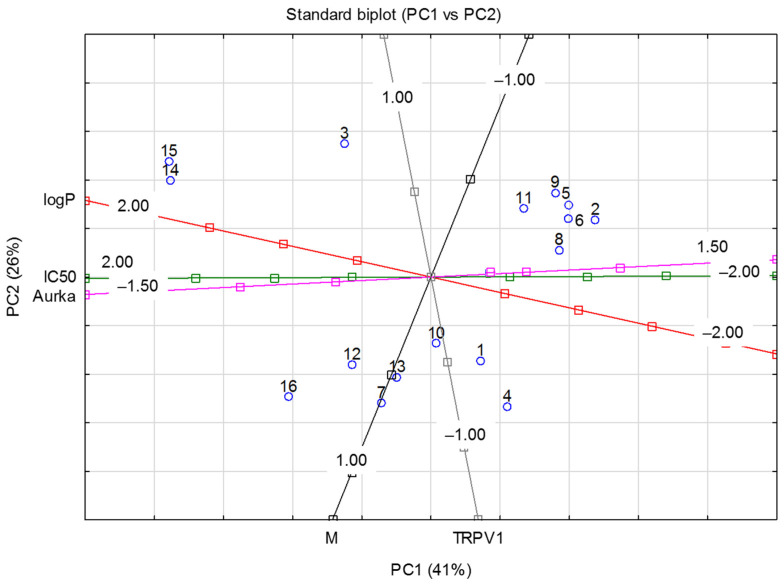
A standard biplot PC1 vs. PC2. Aurka, TRPV1—Total Score values from molecular docking; M—molar mass.

Figure 5 shows a standard PCA biplot where the first principal component (PC1) describes 26% and the second (PC2) describes 41% of the total subset variance. 

These results show an inverse correlation between the affinity to the Aurka and IC50 values. Moreover, the IC50 value can also be connected with the logP value and binding energy to Aurka but not with the affinity to TRPV1 nor molar mass. 

The points corresponding to the molecules with the best activity against both MCF-7 and Aurka are located on the right or central side of the plot. The molecules with the lowest activity are on the left side, which corresponds to the highest IC50 and lowest Total Score, but also higher logP values. This suggests that both the inhibition of Aurora A kinase and the anti-tumor activity against MCF-7 cells require lipophilic properties (preferably a logP value below 3). This corresponds to one of Lipinski’s rule of five, stating that an orally active drug should have an octanol–water partition coefficient [33] (logP) that does not exceed 5. In our study, particles with logP values above 4 have the lowest Total Score values for Aurka and the highest IC50 tested on MCF-7.

The analysis of docked ligands shows that they meet the requirements of the Lipinski rule of 5 [34]. These molecules have no more than five hydrogen bond donors, no more than 10 hydrogen bond acceptors, a molecular mass of less than 500 daltons and an octanol–water partition coefficient (logP) that does not exceed 5. However, in compounds with the lowest docking score, the logP value almost exceeds 5, and ligand **7** has a molecular mass of more than 500 daltons. 

It is possible that the inhibition of the overexpression of Aurka is one of the mechanisms of anticancer activity observed in MCF-7 cell line studies. Chen et al. studied the anticancer activity of capsaicin against breast cancer and concluded that the mechanism could be related to the down-regulation of the FBI-1-mediated NF-κB pathway [35]. The anticancer activity of capsaicin can also be related to the caspase-independent pathway [36]. Thus, the inhibition of Aurka can be one of the new possible anticancer mechanisms of the compounds obtained from peppers. To confirm these results, the in vitro studies are in progress. 

While the ligand–receptor complex obtained from a molecular docking method captures just one moment of the mutual orientation of a ligand and target protein, a more computationally demanding but also much more informative method is molecular dynamics (MD) simulations. This approach enables simulation of the behavior of the modeled system (e.g., ligand–protein complex) in time, allowing to obtain more ligand–protein poses. Consequently, the more poses are being considered, the higher the amount of information provided. Therefore, in this study, we applied both molecular docking and MD simulations to explain the activity profiles of chosen Aurka ligands. 

**Figure 6:**
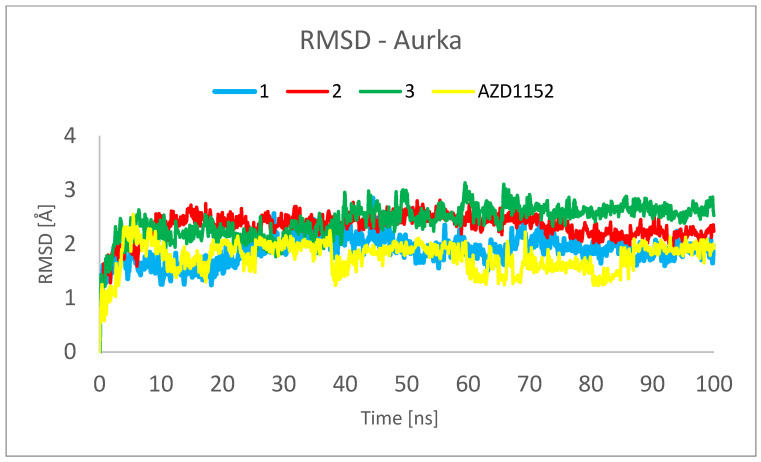
RMSD plot obtained for Cα-atoms of Aurka in the complexes formed between Aurka and chosen ligands during 100 ns MD simulation.

**Figure 7:**
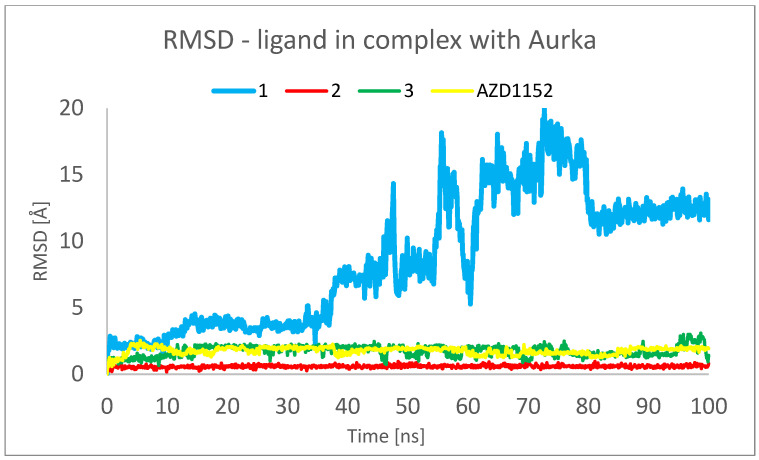
RMSD plots obtained for the studied ligands in the complexes formed between them and Aurka during 100 ns MD simulation.

Figure 6 and Figure 7 show the root mean square deviation of atomic positions (RMSD) plots obtained for Cα-atoms of Aurka (Figure 6) and its chosen ligands (Figure 7) in the complexes formed with protein during 100 ns MD simulation. RMSD analysis can indicate if the simulation has equilibrated—its fluctuations towards the end of the simulation are around some thermal average structure. Usually, changes of the order of 1–3 Å are perfectly acceptable for small proteins. From Figure 6, it can be seen that the RMSD has stabilized itself at the level of c.a. 2.5 Å after c.a. 5 ns of simulations, meaning that the protein molecule in all of the studied complexes is stable from the structural point of view.

**Figure 8:**
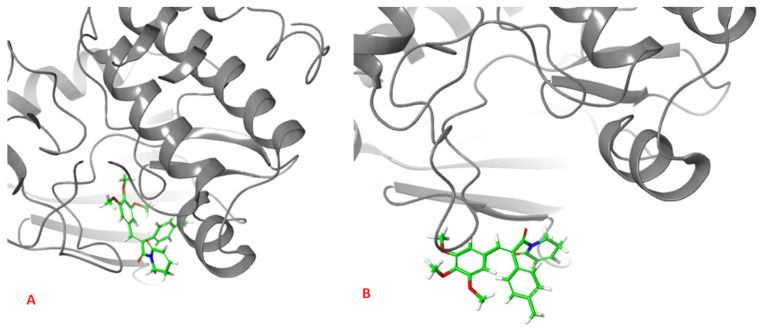
Initial (**A**), and final (**B**), snapshot of the 100 ns MD simulation of the complex formed between **1** and Aurka. During the simulation the dissociation of the complex occurred.

The analysis presented in Figure 7, however, shows totally different behavior of **1** in comparison with other ligands. For **2**, **3** and AZD1152, the RMSD reached the plateau after a few ns of simulation, at the level of 0.5 Å for **2** and 2.0 Å for **3** and AZD1152. However, the complex between the **1** and Aurka, while initially stable during the first 10 ns of simulation, started to dissociate between 15 and 35 ns and dissociated completely during the second half of the simulation period. This is indicated by the large RMSD value, exceeding 15 Å at some points. Additionally, the initial and final snapshots of the complex confirm the observation about its instability and dissociation (Figure 8).

This observation suggests that to better understand the behavior of the complexes formed between Aurka and its ligands, at least 50 ns molecular dynamics simulations should be performed. It is especially important as in the previously published works describing MD simulations of complexes formed between various other ligands and Aurka, significantly shorter total simulation times have been used, i.e., 1 ns [28] and 20 ns [37].

Root mean square fluctuation (RMSF) is a numerical measurement similar to RMSD, but instead of indicating positional differences between entire structures over time, RMSF is a calculation of individual residue flexibility or how much a particular residue moves (fluctuates) during a simulation. Figure S4 (supplementary materials) shows the RMSF plots obtained for Cα-atoms of Aurka in the complexes formed with the studied ligands during 100 ns MD simulation. It can be observed that the tails (N- and C-terminal) fluctuate more than any other part of the protein. The secondary structure elements like alpha helices and beta strands are more rigid than the unstructured part of the protein and thus fluctuate less than the loop regions. In all of the studied complexes, the maximal RMSF per residue was larger than 0.5 Å and was usually not exceeding 3.0 Å, with the exceptions for the termini, both N- and C-. This indicates that no abnormally high dynamic residues were detected, indicating the correctness of the studied model of macromolecules. The major differences in the behavior of Aurka in the studied complexes include the residues 160–170. This fragment is also characterized by the highest RMSD values. Also, solely for the complex with **3**, the large RMSD values were recorded for residues 125–130.

**Figure 9:**
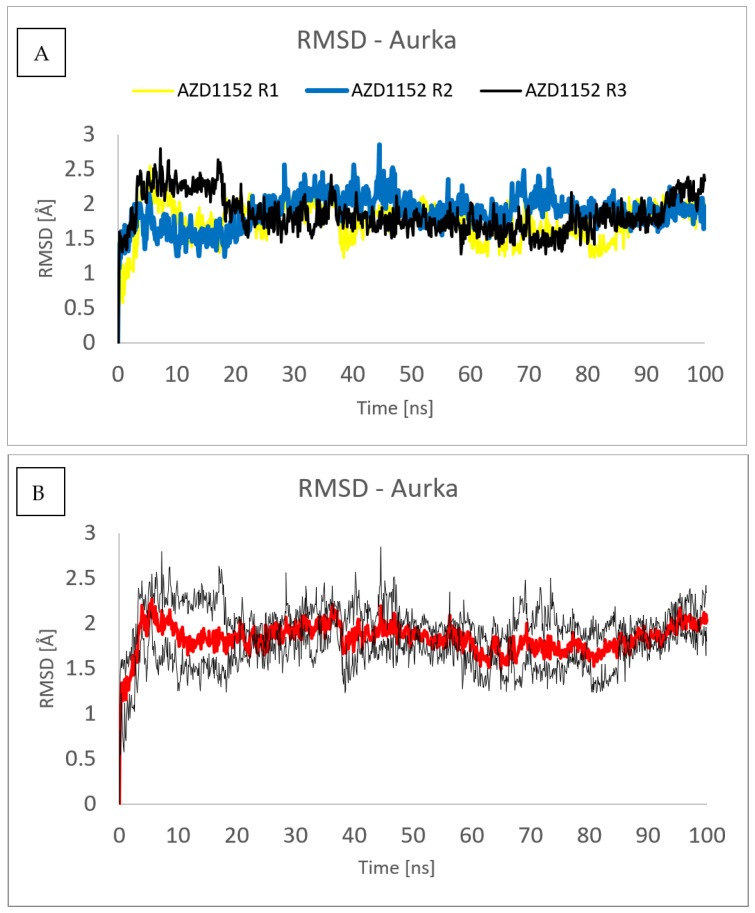
RMSD plot obtained for Cα-atoms of Aurka in the complexes formed between Aurka and AZD1152 during each of the three molecular dynamics trajectories (R1, R2 and R3) (**A**) and average value (red) with the error bars (black) (**B**).

**Figure 10:**
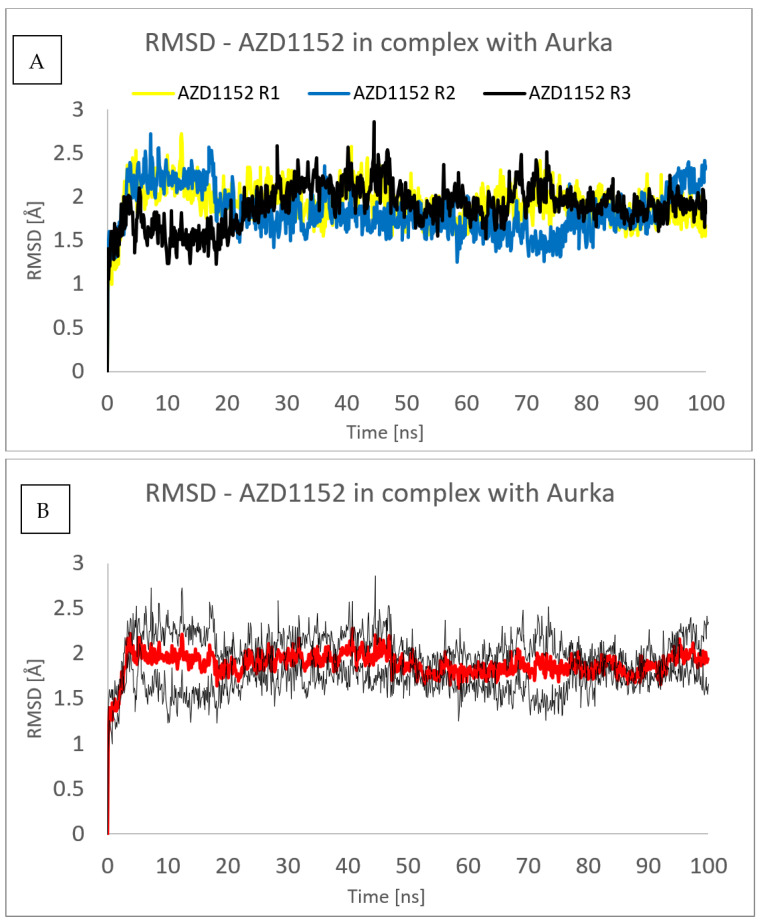
RMSD plot obtained for AZD1152 in the complex formed between Aurka and AZ1152 during each of the three molecular dynamics trajectories (R1, R2 and R3) (**A**) and average value (red) with the error bars (black) (**B**).

To assess the repeatability of results, the molecular dynamics simulations of the complex formed between the Aurka and AZD1152 using the same computational settings have been repeated three times, and the comparisons of the RMSD obtained from those three trajectories are presented in Figure 9 and Figure 10. No major changes have been observed, both for the ligand and the protein.

Figures S5 and S6 (supplementary materials) show the secondary structure (SS) content of the Aurka in its complexes with the studied ligands during the MD simulation. It can be seen that for the vast majority of the residues, their alignment to the particular type of SS remains constant during the simulation, with the exception of the regions characterized by large values of RMSF, which are mostly assigned to unstructured regions of the receptor. In both cases, the SS content remains constant during the simulation, indicating that no major structural transitions can be observed. From the SS analysis point of view, the dynamic behavior of Aurka in all of the studied complexes was similar, with a constant value of SSE at 45% during the whole simulation time.

Specific protein–ligand contacts arise due to multiple weak, low-energy (1–5 kcal/mol), noncovalent interactions such as H-bonds, ionic, and hydrophobic forces at short distance ranges enough for bonding, typically 2.5–3.5 Å. 

**Figure 11:**
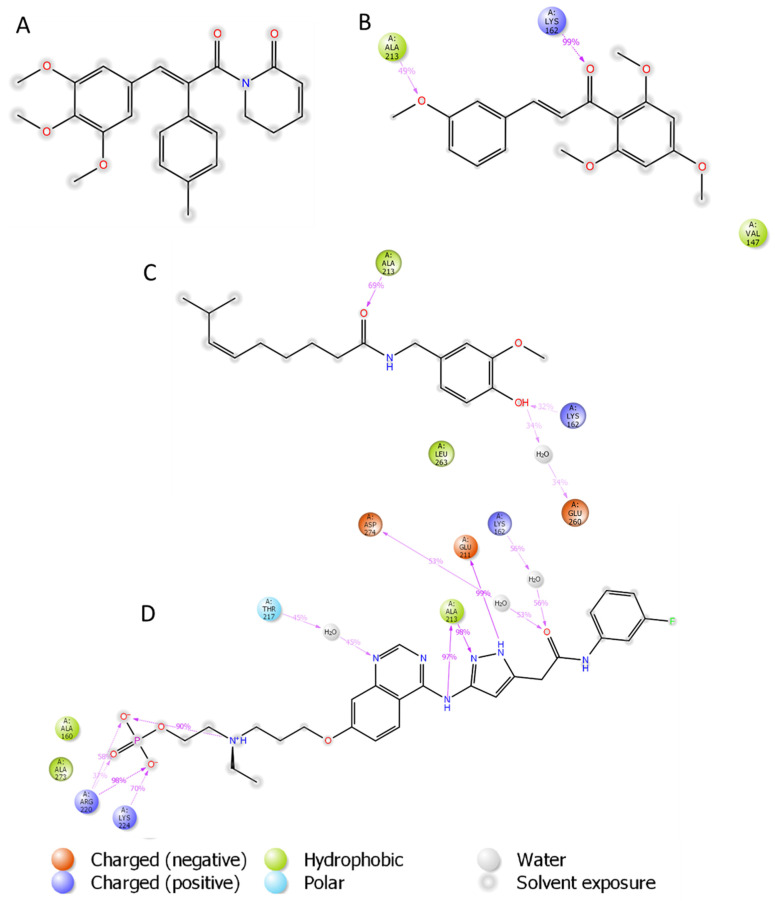
Average percentage of equilibrium simulation time during which Aurka residues maintain contact with the studied ligands. The subsequent figures present the complexes in the order: (**A**) **1**, (**B**) **2**, (**C**) **3**, (**D**) AZD1152.

Protein–ligand interaction diagrams are presented in Figure 11, Figures S7 and S8 (supplementary materials). It should be noted that interactions observed during the MD simulations differ from those obtained from molecular docking. This is due to the presence of water molecules in the system subjected to MD simulations, which were absent in molecular docking. In addition, the simulation enabled the observation of the conformational changes in the ligand and the formation of intermolecular forces between the different functional groups of ligands, mostly in the case of the complex with AZD1152.

Among the residues that form interactions with all of the studies ligands are Arg137, Leu139, Val147, Ala160, Lys162, Leu194, Leu210, Ty212, Ala213, Pro214, Thr217, Arg220, Leu263 and Val279. Only some of those residues, i.e., Val279, form similar interactions in terms of both type and strength, regardless of the ligand. For most of the residues, significant differences can be observed in terms of their interactions with the studied inhibitors. For example, Lys162, which is very important in terms of binding with Aurka, form either direct hydrogen bond type interactions (with **2**) or mixed direct/water bridge mediated H-bonds (with **1** and **3**). For AZD1152, the interaction with Leu162 is partially hydrogen bonding via a water bridge and partially hydrophobic. The importance of this introduction is also shown in the case of the complex of Aurka with **1**. As long as this interaction was present, the complex remained stable. Once this bond was broken, it resulted in complex dissociation. As can be seen, **1** does not form any long (>30% simulation time) lasting interactions with Aurka, which is a consequence of this complex dissociation. Another very important residue, in terms of complex stabilization, is Ala213. When it forms the direct H-bond with the ligand, such as with **2**, **3** or AZD1152, such interaction is strong and stable. On the other hand, if this H-bond is water-mediated, it is eventually broken, resulting in complex dissociation such as in the complex of Aurka with **1**. 

The average number of interactions occurring simultaneously between Aurka and the ligand was 3–4 for the complexes formed with **2**, **3** and 1-until the dissociation occurred (Table 5). A significantly larger number of interactions were observed between AZD1152 and the studied protein, ranging from 9 to 12. In the case of the complex formed between Aurka and its most potent inhibitor, AZD1152, the phosphate group present in this ligand forms multiple strong interactions with Arg220, Lys224, and even one intramolecular H-bond with charged tertiary amine nitrogen. The other nitrogen atoms present in this ligand molecule form multiple H-bonds, either direct (with Glu211 and Ala213) or water mediated (Lys162, Thr217, Asp274). As can be seen in Figure 11, most of those interactions occur during the whole simulation time.

**Figure 12:**
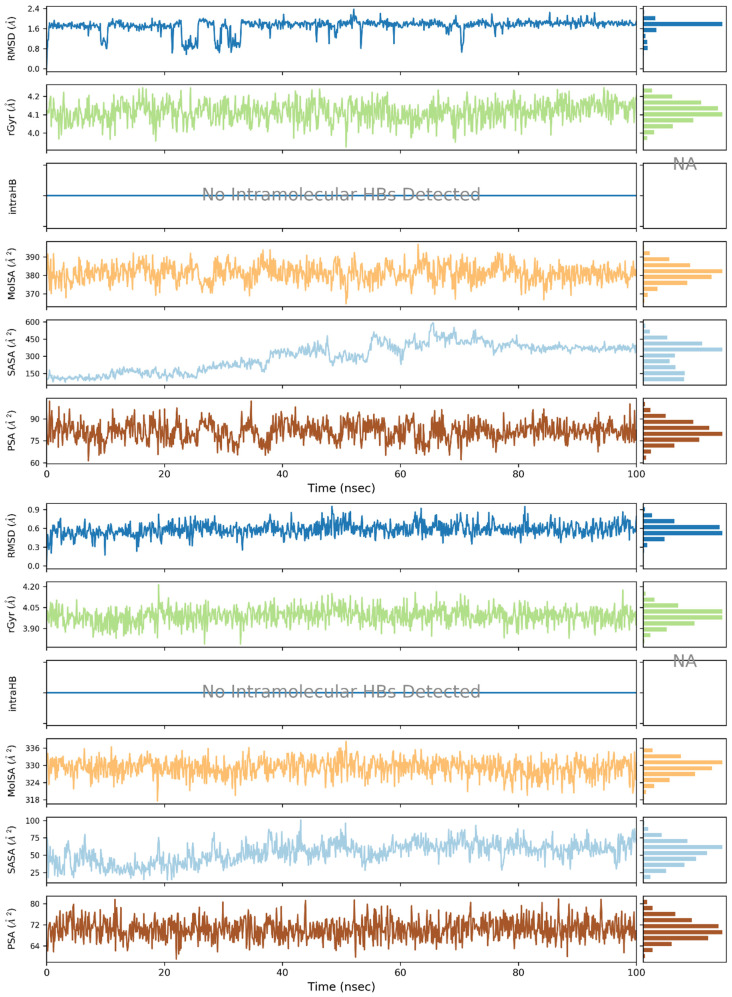

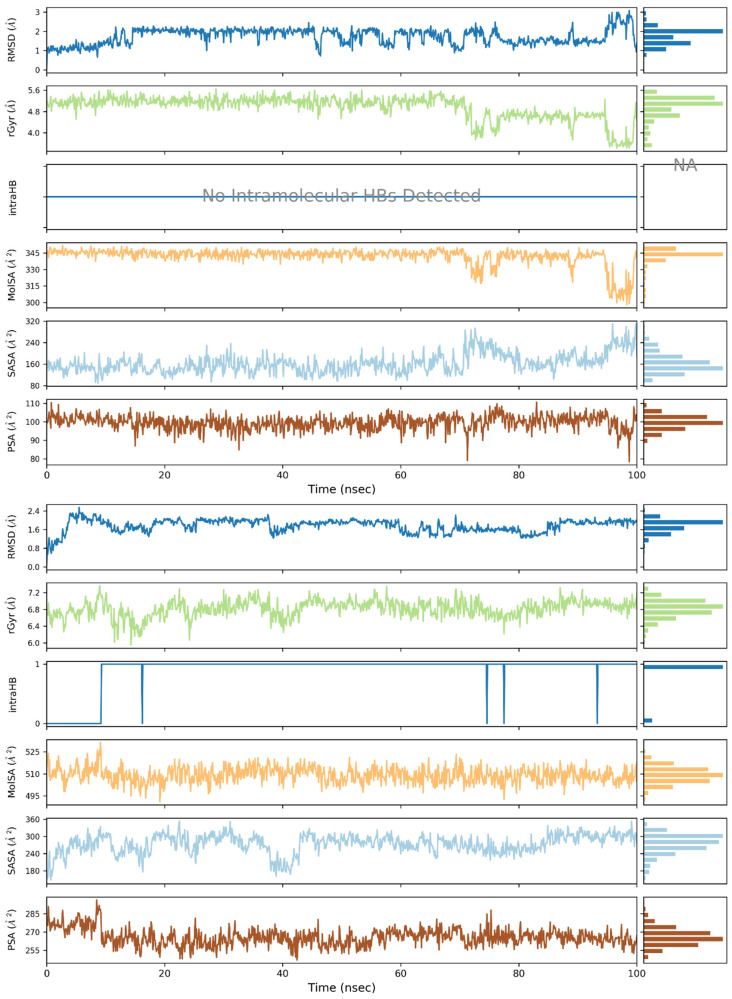
Plots and bar charts of six ligand properties in the complex with Aurka during the simulation (from the bottom): polar surface area (PSA), solvent-accessible surface area (SASA), molecular surface area (MolSA), intramolecular hydrogen bonds (intraHB), radius of gyration (rGyr), and ligand RMSD with respect to the initial conformation. For each property, there is a chart that shows the value of the property as a function of time; to the right, there is a bar chart that shows the proportion of time spent in each of 10 value ranges, divided equally over the range of property values. The subsequent sets of plots and bars are presented for the complexes in the order: **1** (**top**), **2**, **3**, AZD1152 (**bottom**).

To analyze the behavior of the ligand in the form of the complex, Figure 12 was created. The RMSD for ligand was found to be the lowest for **2**, indicating that this ligand conformation is the most stable. In the cases of **1**, **3** and AZD1152, a few conformations of ligand are observed during simulation, which is represented by the different values of RMSD observed during calculations. 

The radius of gyration (rGyr) of the ligand was found to be the largest for AZD1152 as it is also the largest of the studied ligands in terms of molecular size. A decrease in rGyr value during the simulation time, observed for **3**, implies an increase in ligand structure compactness, thereby suggesting decreased flexibility and higher stability. 

In the studied ligands, no intramolecular hydrogen bonds were observed, which is not surprising, taking into consideration their rigid structure and lack of H-bonds donor in the cases of **1** and **2**. An exception to this observation is the behavior of AZD1152, in which a single intramolecular H-bond was formed after 10 ns of simulation, forming between one of the oxygen atoms of the phosphate group and a charged tertiary nitrogen amine atom. Interestingly, this interaction was not present at the beginning of the simulation. This may indicate that either this conformation is more stable under dynamic conditions or the MD was necessary to finding this deeper minimum. 

The polar surface area (PSA) measures the solvent surface area that is produced by polar groups, such as nitrogen and oxygen atoms of the ligand during the simulation [38]. Polar surface area (PSA) was found to oscillate between 70 and 80 A^3^ for **1** and **2**, while it reached 100 A^3^ for **3**. Significantly larger values have been observed for AZD1152, exceeding 250 A^3^. This is not only due to the larger size of this ligand but also as a result of the presence of multiple basic nitrogen atoms and large phosphate group in this ligand.

The SASA refers to the solvent accessibility that provides the quantitative calculation of ligands with protein-implicit water molecules [38]. The studied ligands differ significantly in terms of this parameter. SASA for **2** oscillated between 50 and 75 A^3^, while SASA for **3** was found to be almost two times larger, despite the similar molecular masses of those ligands (328.26 and 305.42 Da, respectively). This was due to the significantly larger flexibility of **3** and the less compact shape of this molecule. Concluding, **3** is characterized by significantly larger flexibility and a less compact shape than **2**; however, during the simulation, a decrease in the flexibility and an increase in compactness of **3** is also observed. In the case of **1**, the 300% increase in SASA during the simulation was caused by the dissociation of the complex and the shift of the ligand from the binding site onto the surface of the protein. This process started after 20 ns of simulation and was completed at 70 ns.

The MolSA measures the water solvent area that ligand molecules acquire. In the MolSA study, the surface area is measured by applying a 1.4 Å probe radius, which is approximately equivalent to the van der Waals surface area and radius of one water molecule [38]. The observed MolSA was found to oscillate around 380, 330, 345 and 510 A^3^, respectively, for **1**, **2**, **3** and AZD1152. The decrease in MolSA for 3 was the direct result of switching the conformation by this ligand, as indicated by the decrease in RoG. 

## 3. Materials and Methods

**Molecular docking validation**

The protocol of molecular docking has been validated in 4 steps:

1. In this study, 12 ligands with known affinity to Aurka expressed as Kd (PubChem AID 624919) were docked to Aurora A kinase using 3 programs (Surflex—Sybyl X 1.2, AutoDock 4.2, and Glide 6.6.), (Table S1, supplementary materials);
2. Kd values were correlated with scoring functions from 3 programs. The best results were obtained by Surflex, so it was recommended for further analysis (Figure S1);
3. To check the correctness of docking, ligand from crystal structure 3H10 was redocked, and the low-energy conformation was compared to conformation from the crystal structure (Figure S2, supplementary materials);
4. Ligands with lower affinity to Aurka (structures and Kd from PubChem AID 624919) were docked to Aurora A using Surflex (Figure S3, supplementary materials).

**Molecular docking**

The set of ligands was selected from compounds obtained from peppers (Piper, Capsicum, and Pimenta), or their analogs with reported anticancer activity on the MCF-7 cell lines. Compounds **1** (piplartine analog), **4** (piplartine analog), **7** (chabamide), **10** (from P. regnelii), **12** (chabamide), **13** (piperine), **16** (from P. regnelii) were obtained from various Piper species. Compounds **6**, **9**, **11** are chalcones from Pimienta methysticum and Pimienta dilatatum) whereas **2**, **5**, **8** are their analogs. Compounds **3** (capsaicin), **14** and **15** (analogs) were obtained from the Capsicum species. Well-known Aurka inhibitors: AZD1152, hesperidin, PHA-73935 [23,24,25] were used for docking study as reference molecules. The structures of ligands were drawn in Sybyl-X 1.2 Sketch (Tripos International). Hydrogens were added and the geometry was optimized using Tripos forcefield (gradient 0.05 kcal/mol × A). The ligand preparation was performed by detecting the root, set number of torsions, and aromaticity criteria using AutoDock Tool [39].

Protein structures were taken from PDB entries 3H10 (Aurka) and 5IS0 (TRPV1). Molecular targets were prepared by removing water, adding hydrogens and performing the optimization using the Sybyl structure preparation tools. Then, protomol (an idealized active site ligand) was defined based on the ligand position in the crystal structures from PDB. The parameters were set as maximum conformations per fragment 20, a maximum number of poses per ligand 20 and maximum RMSD between final poses 0.05. Molecular docking was carried out using Surflex-Dock (SFXC) docking mode. The docking simulations by Autodock 4.2 program [40] applied a Lamarckian genetic algorithm to identify possible binding modes for all ligands. Ligands were prepared for docking by adding charges (gasteiger) and setting roots of torsions. The AutoDock grid box was centered on the ligand from the PDB crystal structure with the size set at 40 × 40 × 40 (x, y and z) and spacing between grid points 0.375 Å. The other parameters of AutoDock were used as default values. The three best compounds against Aurka and a known Aurka inhibitor (AZD1152) were chosen for further MD simulation [41].

The verification of docking was performed by comparing the conformation of capsaicin obtained in our study with that described earlier [32] and its interaction with TRPV1. Similarly, as for Aurka, the comparison was drawn between the results of molecular docking and the molecular mechanics calculation of hesperadin. Conformations of ligands and interactions in the active site were comparable with those described earlier; therefore, we assume that our results are reliable.

**Molecular dynamics (MD)**

Molecular dynamics (MD) simulations were performed using Schrӧdinger Maestro 12.8. version (Schrӧdinger, LLC, New York, NY, USA, 2023). 

### 3.1. Structures Preparation

Three-dimensional crystal structure of Aurka bound to the inhibitor 9-chloro-7-(2,6-difluorophenyl)-N-{4-[(4-methylpiperazin-1-yl)carbonyl]phenyl}-5H-pyrimido[5,4-d][2]benzazepin-2-amine (97B) at 2.20 Å resolution (PDB ID: 3H10) was retrieved from RCBS PDB database [42]. The structure was processed prior to docking using the Protein Preparation Wizard to remove unwanted water molecules and metal ions. This procedure also simplifies multimeric complexes, creates disulfide bonds, assigns bond orders properly, adjusts ionization states, and fixes the orientation of misoriented groups. Hydrogen atoms were added to the protein structure, and standard protonation states at pH 7.0 were used. The preprocessed structure was then optimized and minimized to generate geometrically stable structures. The prepared protein structure was then used for further modeling.

### 3.2. Molecular Docking

#### 3.2.1. Active Site Identification and Grid Generation

Mass center of the co-crystallized ligand (97B) constituted the grid center. A cubic search box was defined with the grid size set large enough to fully accommodate the ligands. Receptor grids were generated with the default parameters for van der Waals scaling factor (1.00) and charge cut-off (0.25) employing the OPLS 2005 force field.

#### 3.2.2. Ligands Preparation

The studied ligands were prepared for docking using LigPrep from the Schrödinger Suite: protonation states were generated at pH 7.4 +/− 2.0, retaining the chiralities. For the geometry optimization, the OPLS 2005 forcefield was used. Other settings of LigPrep remained at default values.

#### 3.2.3. Glide XP-Ligand Docking

Flexible protein–ligand docking was performed using the Grid-based Ligand Docking with Energetics (Glide) module and extra precision (XP) scheme. During the docking, the default 20 poses for the initial Glide docking stage were retained. Glide score (GScore) was calculated as GScore = 0.065 × vdW + 0.130 × Coul + Lipo + Hbond + Metal + BuryP + RotB + Site, wherein vdW: van der Waals energy; Coul: Coulomb energy; Lipo: Lipophilic term; Hbond: Hydrogen bonding; Metal: Metal-binding term; BuryP: Buried Polar groups’ penalty; RotB: Penalty for rotatable bonds that have been frozen; Site: active site polar interactions. Emodel combines GlideScore, the nonbonded interaction energy, and, for flexible docking, the excess internal energy of the generated ligand conformation.

#### 3.2.4. MM-GBSA Calculations

Calculation of binding free energy (ΔGbind) values was exploited to estimate in silico ligand binding affinities. For accurate calculation of binding free energies, Molecular Mechanics/Generalized Born Surface Area (MM/GBSA) rescoring method was used. For this purpose, the Prime MM/GBSA module was utilized.

MM/GBSA rescoring was performed for initial ligand-docked poses with best scoring functions. The free energy changes of during protein ligand interactions were calculated with the use of OPLS 2005 force field and the VSGB solvent model.

Binding free energy values were calculated according to the following equation:

MM/GBSA ΔGbind = Gcomplex (optimized) − (Gprotein (optimized) + Gpeptide (optimized))

Free energy of each state, i.e., of complex, protein and peptide, was estimated by accounting for molecular mechanics energies, solvation energies, and entropic terms, as follows:

G = Gint + GCoulomb + GvdW + GGB + Glipo − TS

where Gint, GCoulomb, and GvdW are standard MM energy terms for bond (covalent, angle and dihedral), Coulomb (electrostatic) and van der Waals interactions, GGB and Glipo are polar and non-polar (lipophilic) contributions to the solvation free energies, while T is an absolute temperature and S is an entropy value. Polar contribution (GGB) was calculated using Generalized Born model, while non-polar contribution (Glipo) was estimated based on the solvent accessible surface area (SASA).

### 3.3. Molecular Dynamics (MD) Simulations

The obtained ligand–receptor complexes with the best Gscores constituted an input for MD simulations. For that purpose, the Desmond System Builder module was used. The ligand–protein complexes were put in the orthorhombic boxes. The systems were solvated with a water, using TIP3P water model [43], with a buffer distance of 10 Å. In each case, the system was neutralized by the addition of the appropriate number of Cl-ions. The systems were subjected to steepest descent minimization with Desmond’s default protocol prior to performing MD simulations.

The relaxation protocol consists of eight stages that included minimization with restraints on solute heavy atoms, minimization without any restraints, simulation with heating from 0 K to 300 K, H_2_O barrier and gradual restraining, simulation under NPT equilibration with H_2_O barrier with heavy atoms restrained, NPT equilibration of solvent and lipids, simulation under the NPT ensemble with protein heavy atoms restraint reduced from 10.0 to 2.0 kcal/mol, NPT equilibration with Cα atoms restrained at 2 kcal/mol, and simulation for 1.5 ns under the NPT ensemble with no restraints.

After relaxation, an unrestrained simulation run was performed for 100 ns for each system. The simulations were performed under the NPT ensemble using the Nose–Hoover thermostat to maintain a constant temperature of 300 K and isotropic Martyna–Tobias–Klein barostat to maintain the pressure at 1 atm. The short-range Coulombic interactions were analyzed with a cut-off value of 9.0 Å using the short-range method. A time-reversible reference system propagator algorithm (RESPA) integrator was used with a time step of 2.0 fs. The trajectories were saved at 100 ps intervals for analysis. After simulations were performed, RMSD, RMSF, and protein–ligand contacts were evaluated using a Simulation Interaction Diagram from the Schrödinger Suite. Interactions occurring in each frame of the performed simulations were encoded in the form of interaction fingerprints (IFPs).

The LogP (octanol–water coefficient) and molar mass values were calculated in HyperChem 7.5 for each ligand. PCA was evaluated using Statistica 10 (StatSoft Inc., Tulsa, OK, USA).

## 4. Conclusions

The conducted research showed that the compounds contained in various varieties of pepper can inhibit Aurora A kinase. Molecular docking allowed us to select the three best compounds showing kinase activity. Analysis of the docking results suggests that there is probably no relationship between the strength of the interaction with the TRPV1 receptor and the Aurora A kinase. The molecular dynamics study confirms that 2 and 3 form a stable complex with Aurora A. Our results show that the duration of the MD simulations is significant for the reliability of the results. In the case of short simulations, it would not be possible to detect that 1 forms an unstable complex. The best compounds selected from our study will be used in subsequent analyses using in vitro activity assays.

## Figures and Tables

**Table 2:** Docking scores for the best poses of selected ligands using Surflex and Glide and values of calculated binding free energies MM/GBSA ΔG_bind_ and its contributions, ΔG_Coulomb_, ΔG_vdW_ are standard MM energy terms for Coulomb (electrostatic) and van der Waals interactions, ΔG_GB_ and ΔG_lipo_ are polar and non-polar (lipophilic) contributions to the solvation free energies. All the values are in kcal/mol.

Ligand	Surlfex Total Score	Glide XP Docking Score	MM/GBSA ΔG_bind_	ΔG_Coulomb_	ΔG_vdW_	ΔG_GB_	ΔG_lipo_
1	6.752	−4.734	−72.94	−15.04	−42.91	25.72	−45.08
2	6.592	−6.816	−62.84	−8.45	−36.93	14.42	−44.98
3	6.025	−8.075	−67.72	−13.26	−33.08	16.37	−35.49
4	6.327	−4.935	−71.80	−8.01	−47.52	20.19	−47.49
5	6.058	−5.736	−63.16	−14.29	−41.45	19.12	−41.89
6	5.979	−7.877	−70.37	−19.78	−35.17	17.58	−33.26
7	5.931	−7.982	−92.73	−13.48	−58.25	23.40	−47.48
8	5.918	−6.785	−62.10	−12.69	−35.60	15.95	−39.51
9	5.813	−7.601	−69.19	−11.39	−42.49	15.87	−38.31
10	5.753	−8.137	−69.28	−13.59	−33.85	15.61	−39.21
11	5.685	−7.526	−62.28	−14.55	−35.51	17.35	−32.10
12	5.499	−7.096	−89.38	−19.83	−48.98	23.12	−46.36
13	5.191	−5.846	−67.96	−6.34	−38.47	15.09	−41.65
14	5.047	−4.895	−50.38	50.45	−42.17	−34.91	−22.17
15	4.894	−7.568	−68.11	33.84	−39.84	−31.17	−33.02
16	4.332	−8.055	−66.23	−14.67	−31.60	13.11	−34.17

**Table 3:** Docking scores for the best pose of selected ligands using AutoDock.

Ligand	BE	IC	IE	TIE	TE	UE
1	−8.97	263.81 nM	−10.76	−0.97	1.79	−0.97
2	−7.22	5.09 μM	−9.31	−0.65	2.09	−0.65
3	−7.13	13.97 μM	−10.11	−1.32	2.98	−1.32
4	−6.30	24.29 μM	−8.38	−1.65	2.09	−1.65
5	−6.08	35.22 μM	−8.16	−1.13	2.09	−1.13
6	−7.66	2.44 μM	−9.75	−1.21	2.09	−1.21
7	−7.57	2.82 μM	−9.06	−2.35	1.49	−2.35
8	−6.62	14.09 μM	−8.41	−0.63	1.79	−0.63
9	−7.09	6.33 μM	−9.18	−1.14	2.09	−1.14
10	−8.3	823.59 nM	−9.49	−0.69	1.19	−0.69
11	−7.66	2.44 μM	−9.45	−1.18	1.79	−1.18
12	−6.79	10.62 μM	−8.28	−2.54	1.49	−2.54
13	−7.90	1.61 μM	−8.8	−0.39	0.89	−0.39
14	−6.3	4.45 μM	−8.79	−1.51	1.49	−1.51
15	−5.91	18.64 μM	−8.0	−1.57	2.09	−1.57
16	−5.84	19.62 μM	−7.04	−1.02	1.19	−1.02

BE—binding energy; IC—inhibition constant; IE—intermolecular energy; TIE—total internal energy; TE—torsional energy; UE—unbound energy.

**Table 4:** Total Score and IC50 values of selected known Aurka inhibitors [24,25,26].

Inhibitor	Total Score	IC50 [nM] [27]
AZD1152	8.043	0.37
Hesperadin	5.817	250
PHA-73935	6.925	13

**Table 5:** The list of residues in the active site of Aurka interacting with ligands.

Ligand	H-Bonding Residues	H-Bonding Water Mediated Residues	Hydrophobic Interacting Residues
(2)	Ala213, Lys162		Val147
(3)	Ala213, Lys162,	Glu260	Leu253
AZD1152	Ala213, Glu211, Lys224, Arg220	Lys162, Asp274, Thr217	Ala273, Ala160

## Data Availability

Data is contained within article and Supplementary Materials.

## Supplementary Materials

The following supporting information can be downloaded at: https://www.mdpi.com/article/10.3390/ph16111539/s1, Table S1: Ligands used in validation protocol with Kd value and scoring functions from 3 programs. Kd are expressed as PubChem Standard Value (PubChem AID 624919); Table S2: Docking Total Score values for the best poses of selected ligands using Surflex to TRPV1; Figure S1: Linear regressions between Kd values and docking scores from 3 programs (A. Surflex, B. AutoDock, C. Glide), R2—coefficients of determination (A. 0.67, B. 0.41, C. 0.21); Figure S2: Comparison of the conformation of the inhibitor from the 3H10 crystal structure (blue) and this compound docked to Aurka (red) in the active site of the tested protein; Figure S3: Results of docking of compounds with lower affinity (Kd > 10 PubChem 624919) to Aurka; Figure S4: RMSF plot obtained for Cα-atoms of Aurka in the complexes formed between ligands and Aurka during 100 ns MD simulation; Figure S5: Secondary structure content (SSE, %) of the Aurka as a function of residue number; Figure S6: The secondary structure content of Aurka as a function of time; Figure S7: Protein–ligand contacts chart showing fraction of the simulation time during the studied ligands were in contact with Aurka residues; Figure S8: The average number of interactions (total-up, per specific residue-bottom) over the simulation time for Aurka.
