# Supplementary material for: Searching for Natural Aurora a Kinase Inhibitors from Peppers Using Molecular Docking and Molecular Dynamics
Source: Pharmaceuticals (Basel). 2023 Oct 31;16(11):1539. doi: 10.3390/ph16111539 (PMC10674409; doi:10.3390/ph16111539)
Supplement: Supplementary file 1 [file pharmaceuticals-16-01539-s001.zip › pharmaceuticals-2636644-supplementary.pdf]

Supplementary materials

Table S1. Ligands used in validation protocol with Kd value and scoring functions from 3 programs. Kd are expressed as PubChem Standard Value (PubChem AID 624919).

Structure	Symbol	Kd	Total Score Surflex	Binding Energy AutoDock	XP Score Glide
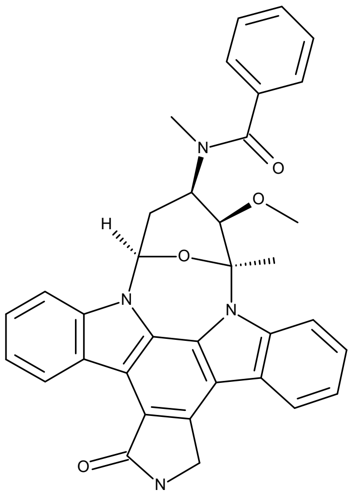	CHEBI 63452	0.12	7.19	-10.91	-10.12
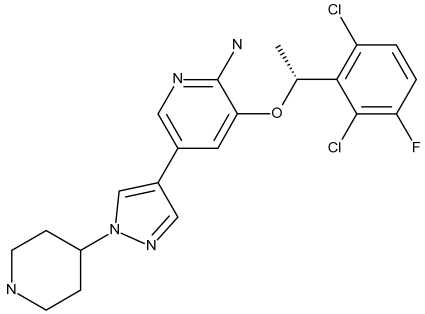	CHEBI 64310	0.26	6.76	-10.47	-9.39

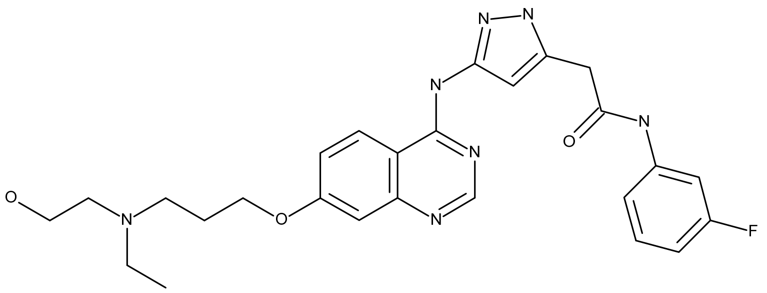	AZD 1152	0.4	7.36	-8.58	-8.99
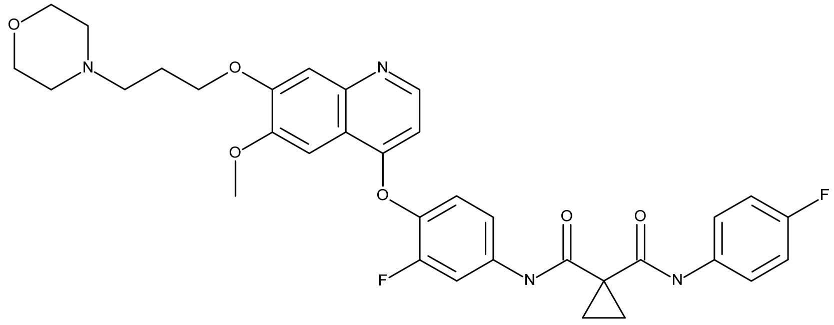	EXEL 2880	0.44	6.60	-9.41	-4.65
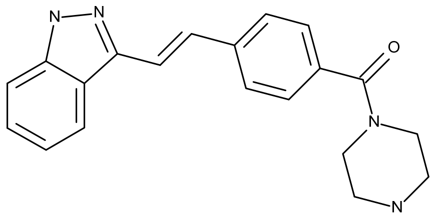	KW 2449	0.65	6.57	-9.55	-8.81
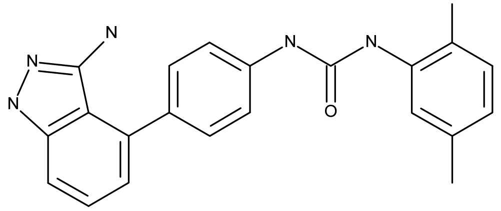	A741 439	1.6	6.25	-9.65	-8.73

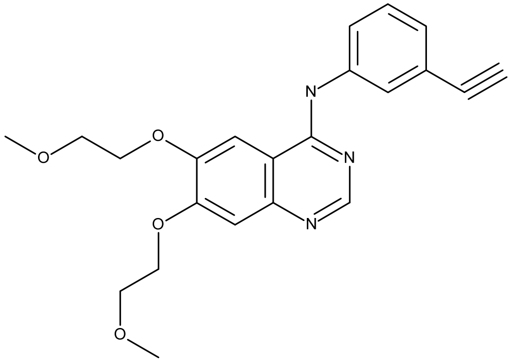	CHEBI 114785	2.2	7.50	-7.45	-8.56
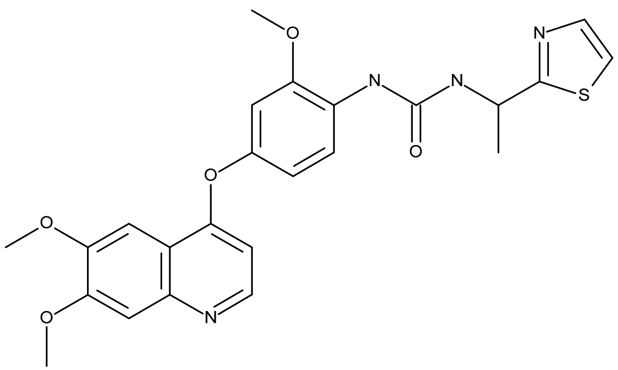	KI 20227	2.3	5.77	-9.57	-9.44
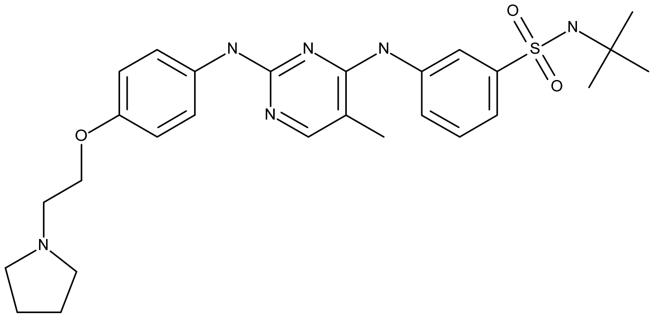	Fedratinib	4.4	6.93	-8.09	-6.73

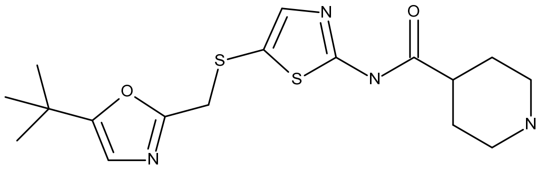	BMS 387032	7.4	5.36	-7.19	-7.70
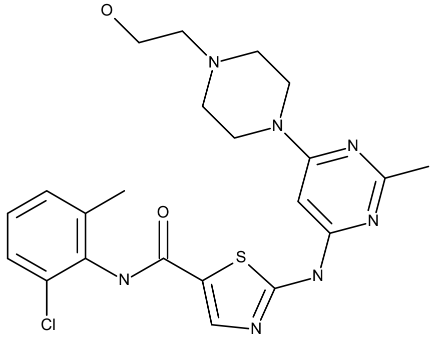	CHEBI 49375	9.3	5.20	-8.1	-7.58
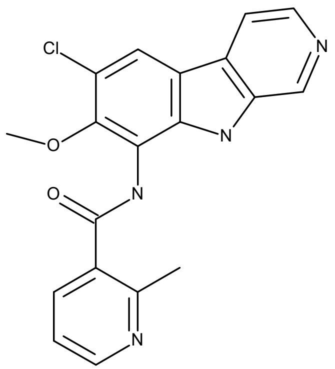	ML 120	>10	2.89	-8.4	-5.71

Table S2. Docking Total Score values for the best poses of selected ligands using Surflex to TRPV1.

No	TRPV1 Total Score
1	5.023
2	4.872
3	5.830
4	4.250
5	5.284
6	4.886
7	4.760
8	3.944
9	5.500
10	4.824
11	4.867
12	5.694
13	4.195
14	4.966
15	5.513
16	3.642

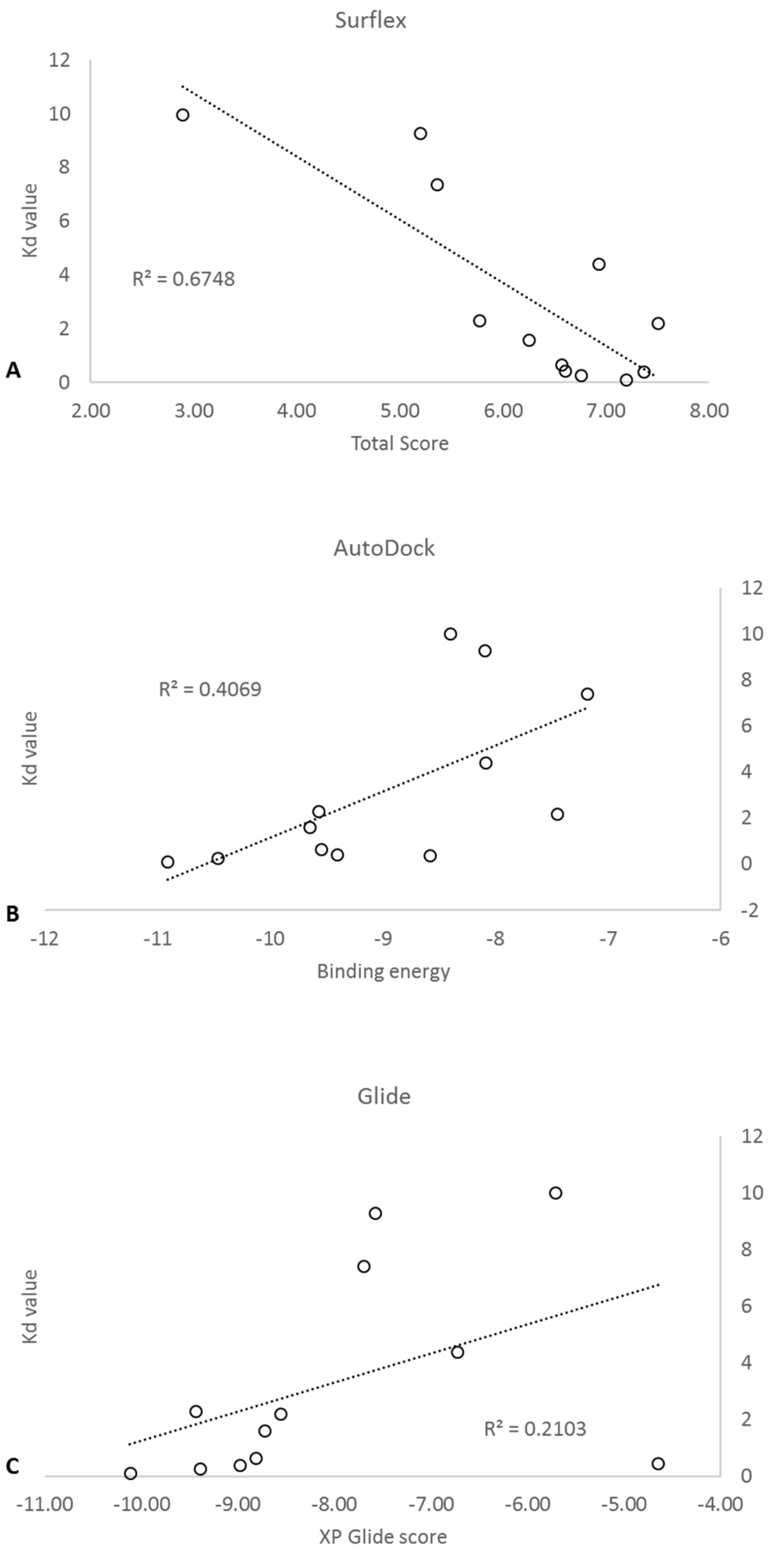

Figure S1. Linear regressions between Kd values and docking scores from 3 programs (A. Surflex, B. AutoDock, C. Glide), R^2 – coefficients of determination (A.0.67, B. 0.41, C. 0.21).

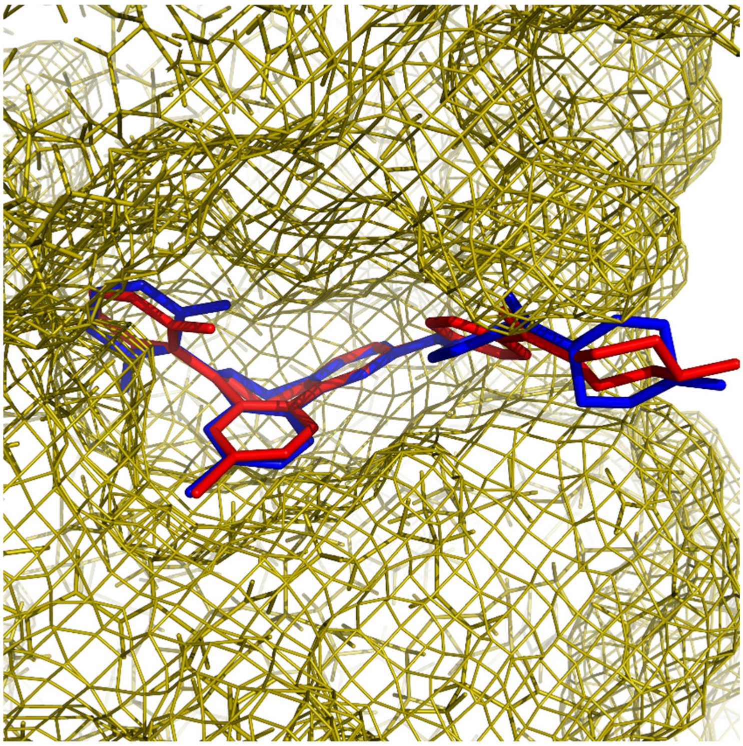

Figure S2. Comparison of the conformation of the inhibitor from the 3H10 crystal structure (blue) and this compound docked to Aurka (red) in the active site of the tested protein.

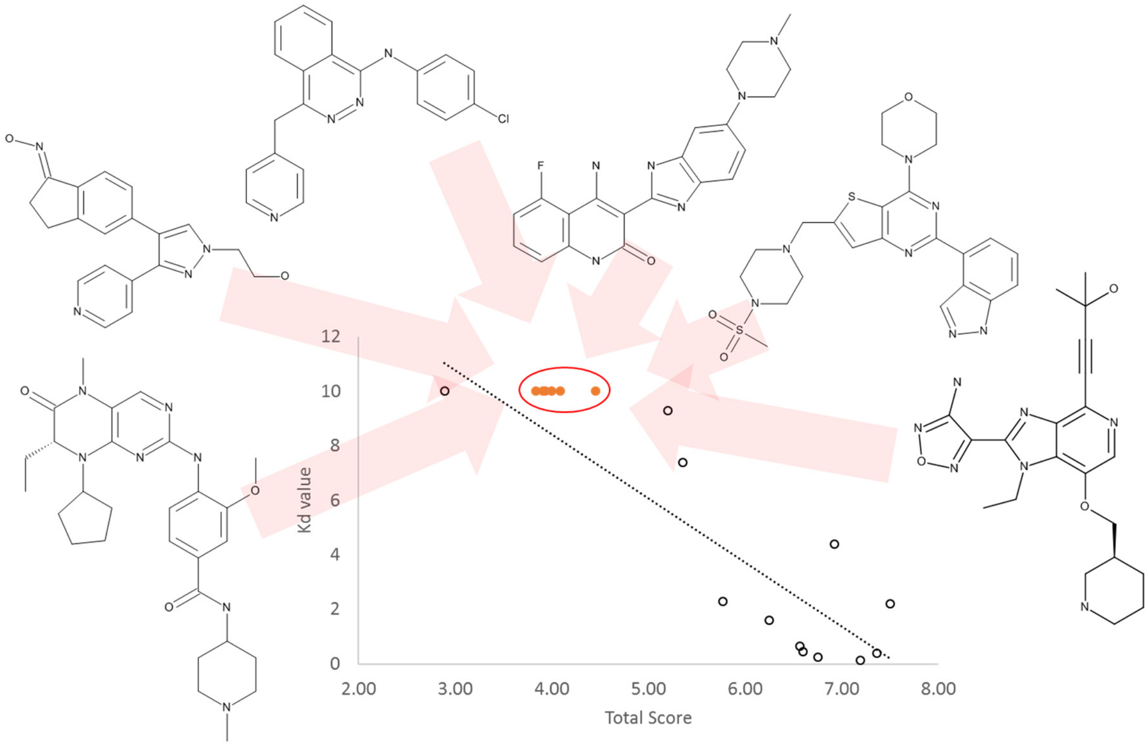

Figure S3. Results of docking of compounds with lower affinity (Kd>10 PubChem 624919) to Aurka.

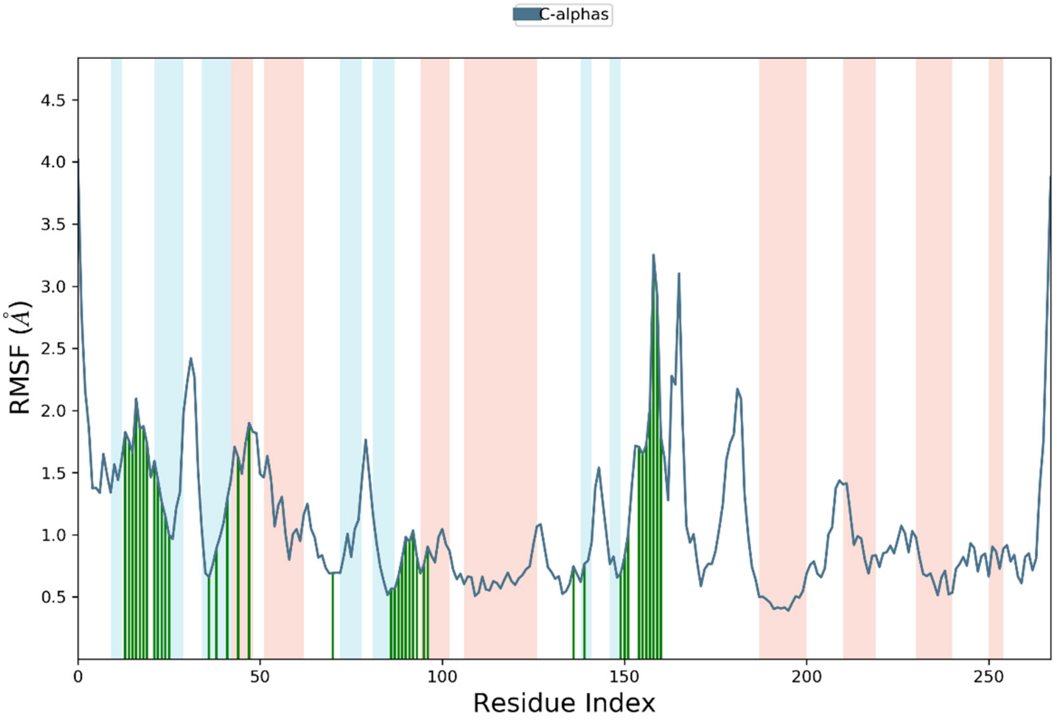
C-alphas
RMSF (Å)
Residue Index
0.5
1.0
1.5
2.0
2.5
3.0
3.5
4.0
4.5
0
50
100
150
200
250

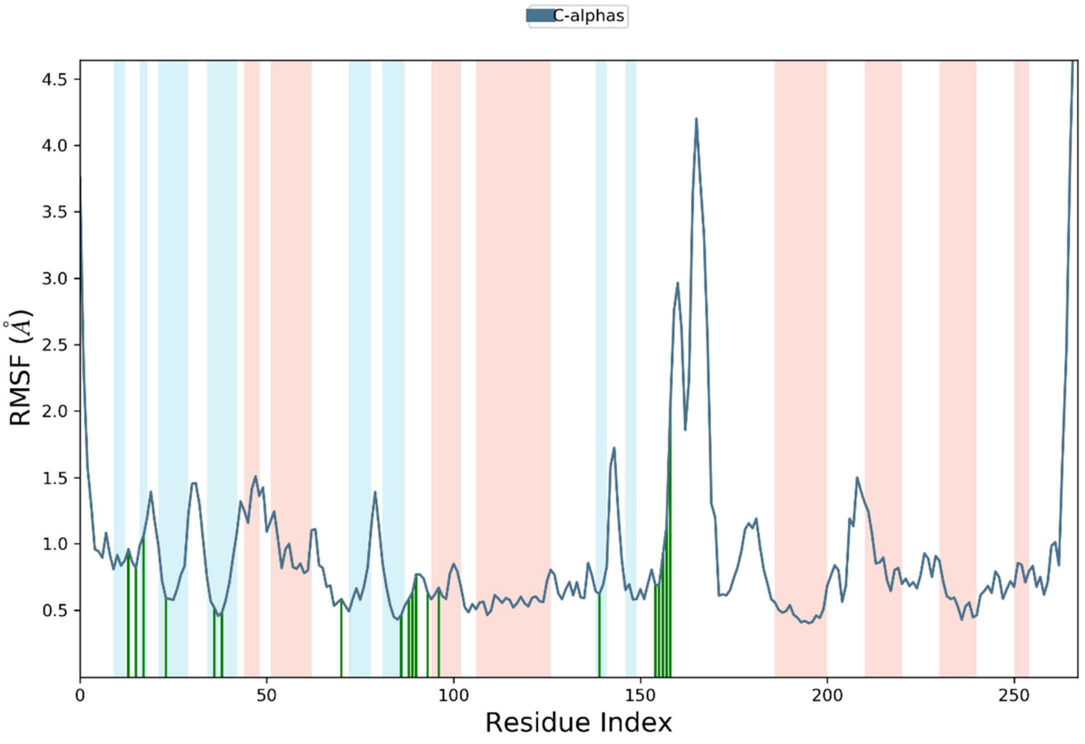
C-alphas
RMSF (Å)
Residue Index
0.5
1.0
1.5
2.0
2.5
3.0
3.5
4.0
4.5
0
50
100
150
200
250

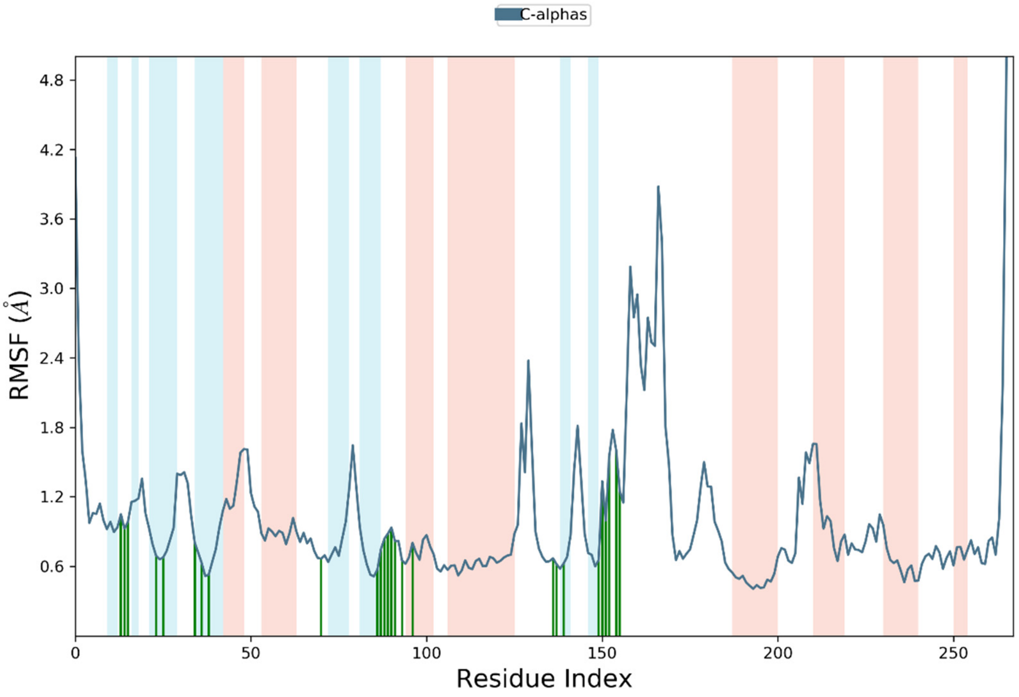

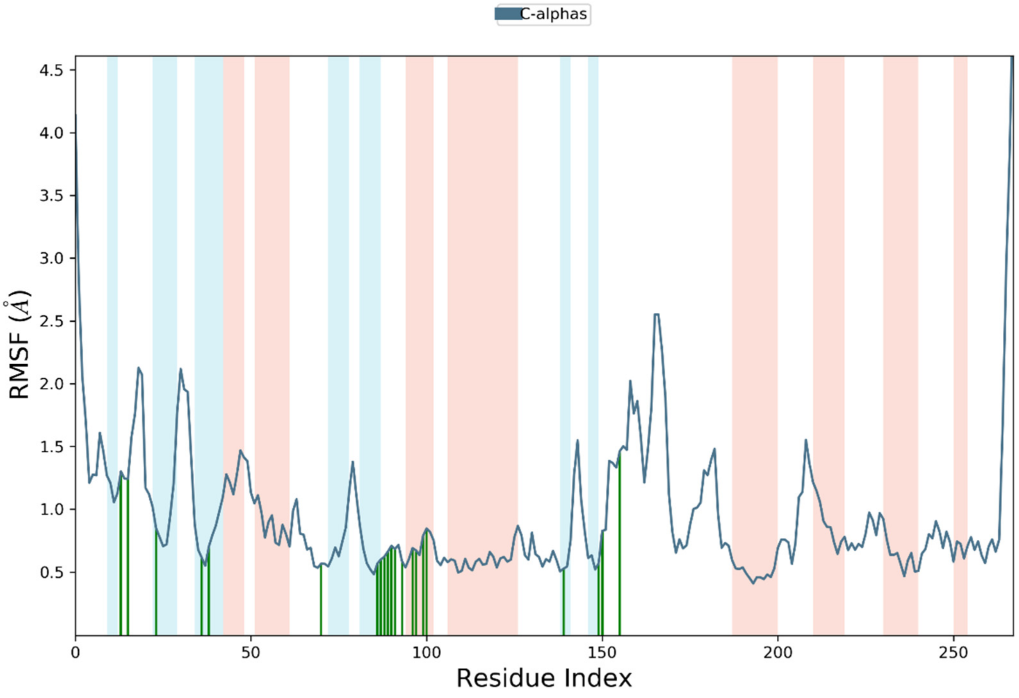

Figure S4. RMSF plot obtained for Cα-atoms of Aurka in the complexes formed between ligands and Aurka during 100 ns MD simulation. The secondary structure elements: strand (blue) and helix (orange). Green lines indicate the residues that form ligand-protein interactions. The subsequent figures present the complexes in the order: **1** (top), **2**, **3**, AZD1152 (bottom).

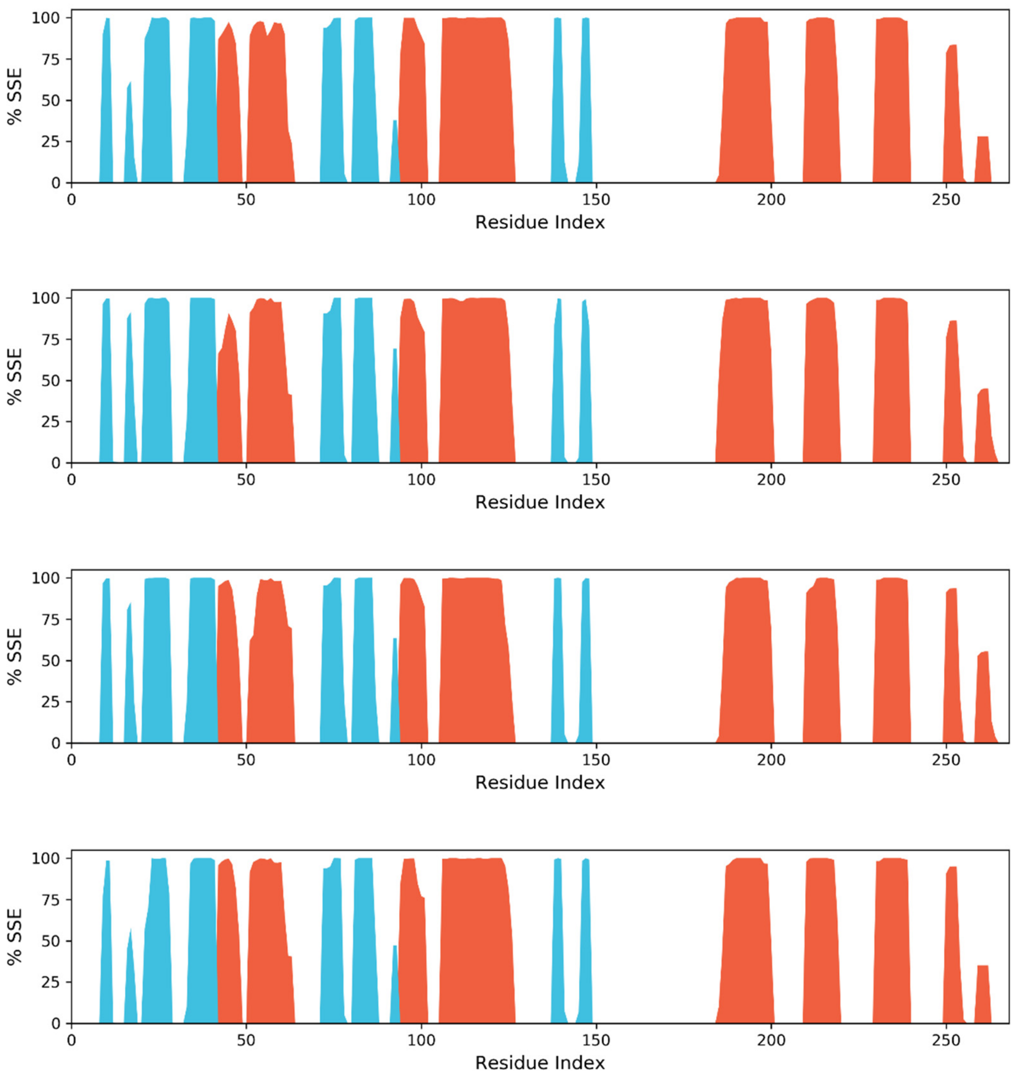

Figure S5. Secondary structure content (SSE, %) of the Aurka as a function of residue number. The secondary structure elements: strand (blue) and helix (orange). The subsequent figures present the complexes in the order: **1** (top), **2**, **3**, AZD1152 (bottom).

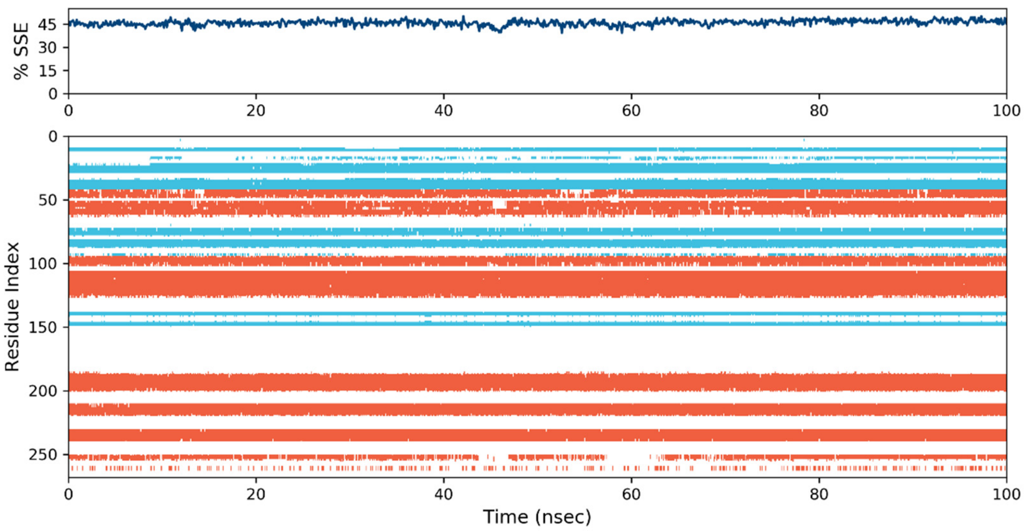
% SSE
45
30
15
0
0
20
40
60
80
100
Residue Index
0
50
100
150
200
250
Time (nsec)

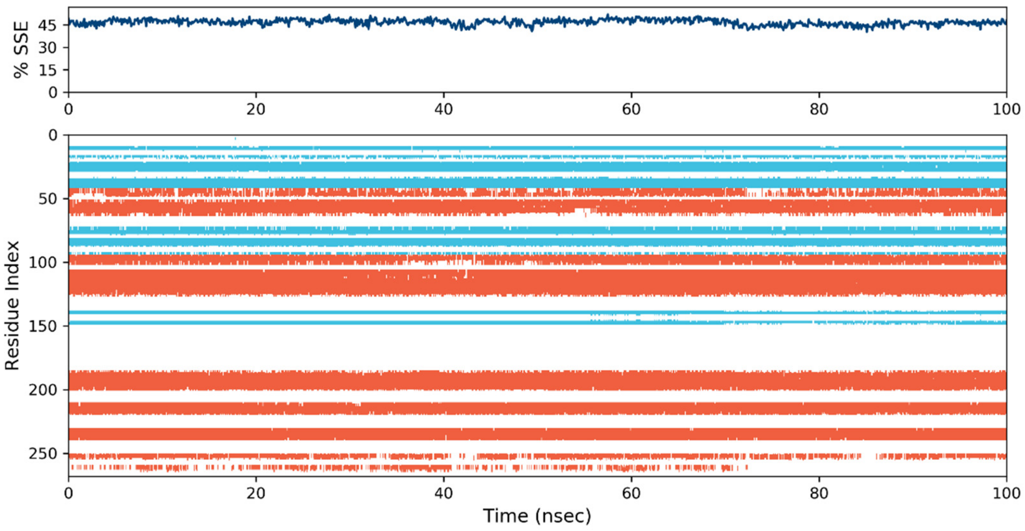
% SSE
45
30
15
0
0
20
40
60
80
100
Residue Index
0
50
100
150
200
250
Time (nsec)

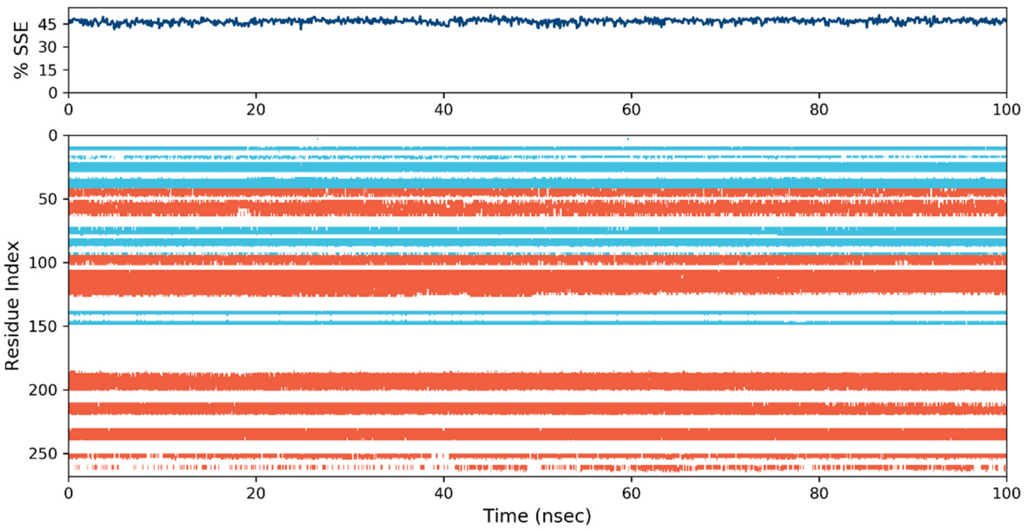
% SSE
45
30
15
0
0
20
40
60
80
100
Residue Index
0
50
100
150
200
250
Time (nsec)

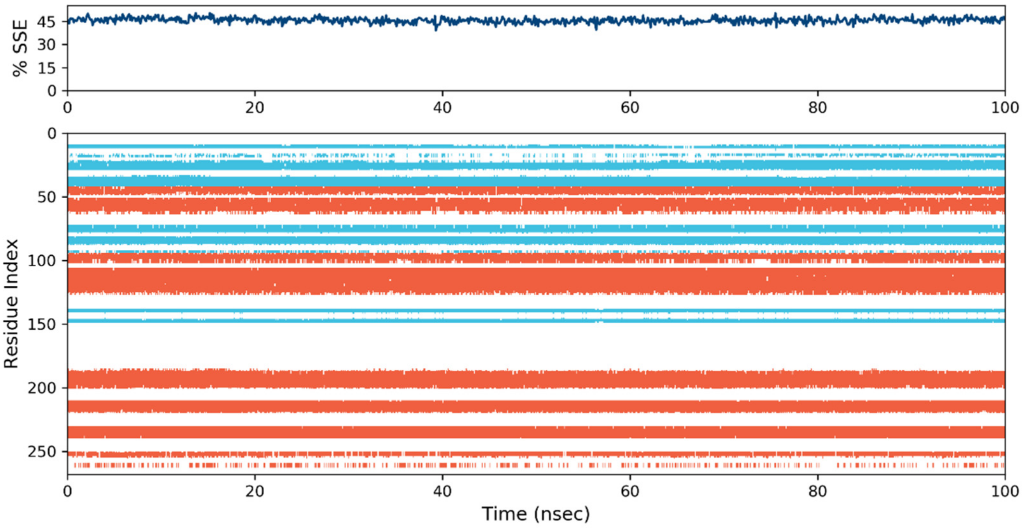

Figure S6. The secondary structure content of Aurka as a function of time. The upper plot shows the secondary structure content as a function of time as the total helix+strand and the lower plot presents the secondary structure content as a function of time of residue index. The subsequent figures present the complexes in the order: **1** (top), **2**, **3**, AZD1152 (bottom).

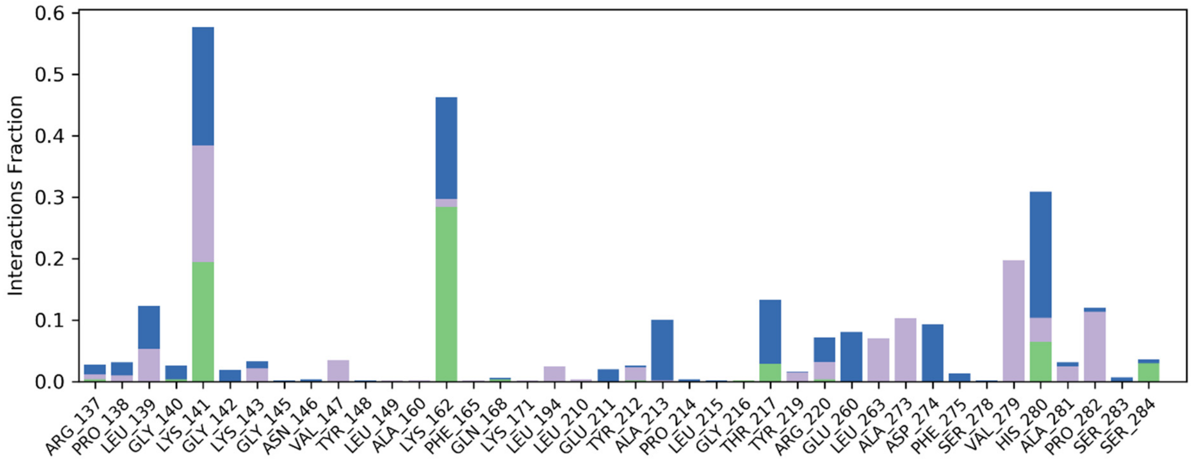

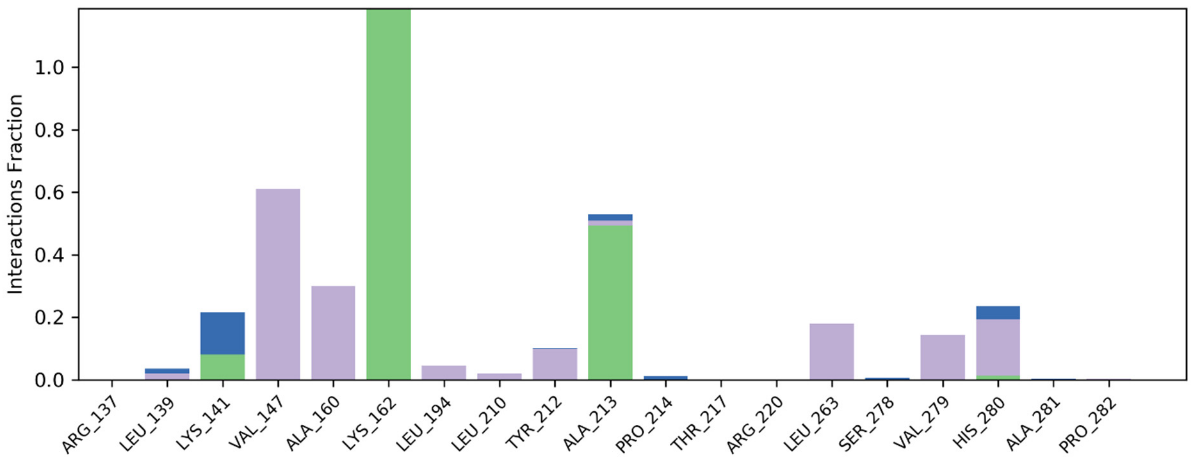

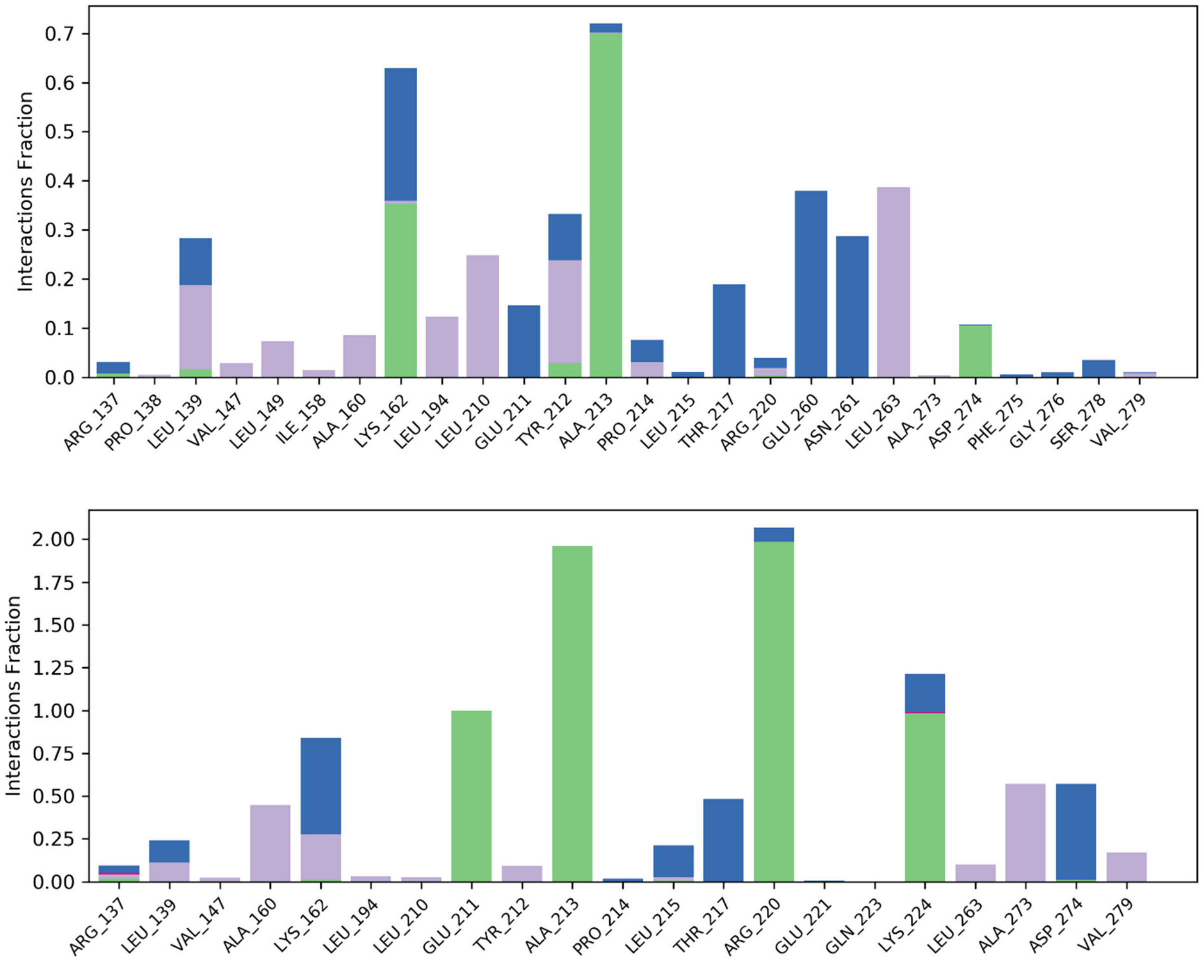

Figure S7. Protein-ligand contacts chart showing fraction of the simulation time during the studied ligands were in contact with Aurka residues. Blue color represents the interactions that involve hydrogen bonding via a water bridge molecule. The geometric criteria are a H-A distance less than 2.7Å, a D-H-A angle greater than 110°, and a H-A-X angle greater than 80° for the hydrogen bonds to the bridging water. Green color shows hydrogen bonds, defined by distances and angles of the D-H...A-X atom arrangement: a H-A distance less than 2.8Å, a D-H-A angle greater than 120°, and a H-A-X angle greater than 90°. Purple color indicates hydrophobic interactions, broken down into three types: pi-pi stacking—two aromatic groups stacked face-to-face, with distance between centroids less than 4.4Å and angle between planes less than 30°, or face-to-edge, with distance between centroids less than 5.5Å and angle between planes greater than 60°; pi-cation—aromatic and charged group centroids within 4.5Å, and general—hydrophobic side chain within 3.6Å of ligand aromatic or aliphatic carbon. The subsequent figures present the complexes in the order: **1** (top), **2**, **3**, AZD1152 (bottom).

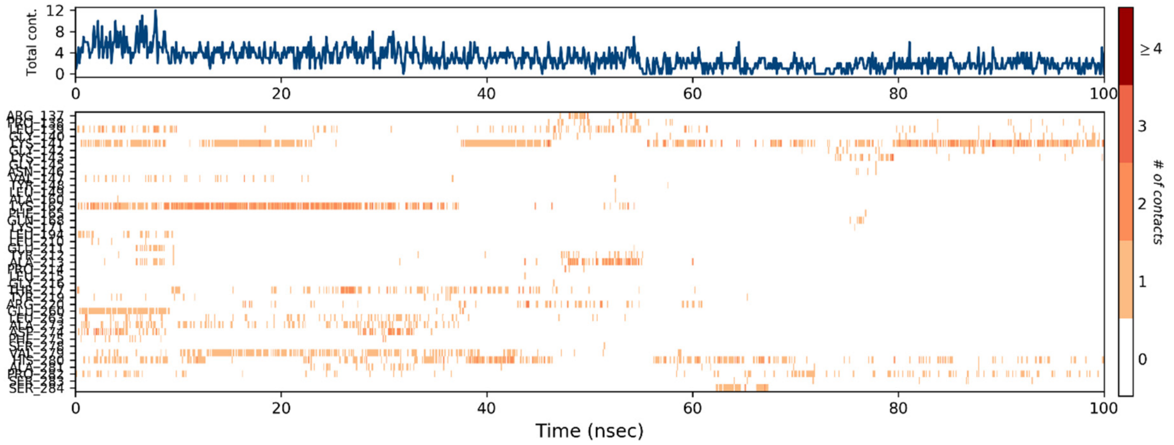
Total cont.
12
8
4
0
0
20
40
60
80
100
Time (nsec)
≥4
3
2
1
0
# of contacts

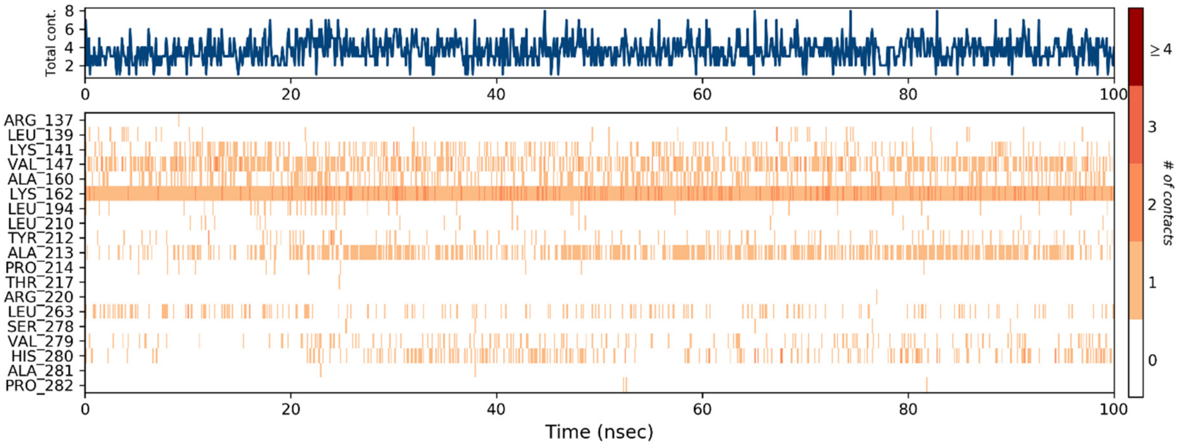
Total cont.
8
6
4
2
0
20
40
60
80
100
ARG_137
LEU_139
LYS_141
VAL_147
ALA_160
LYS_162
LEU_194
LEU_210
TYR_212
ALA_213
PRO_214
THR_217
ARG_220
LEU_263
SER_278
VAL_279
HIS_280
ALA_281
PRO_282
Time (nsec)
≥4
3
2
1
0
# of contacts

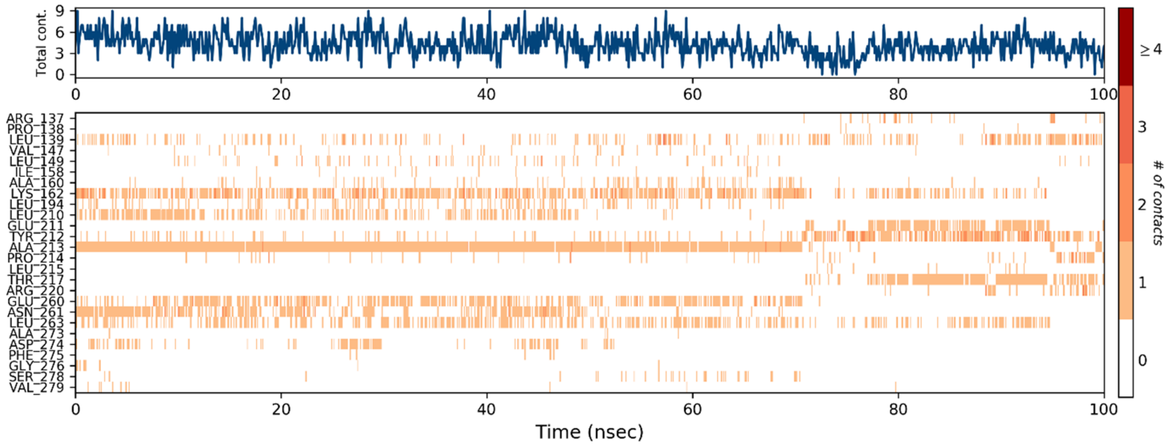
Total cont.
9
6
3
0
0
20
40
60
80
100
ARG_137
PRO_138
LEU_139
VAL_147
LEU_149
ILE_158
ALA_160
LYS_162
LEU_194
LEU_210
GLU_211
TYR_212
ALA_213
PRO_214
LEU_215
THR_217
ARG_220
GLU_260
ASN_261
LEU_263
ALA_273
ASP_274
PHE_275
GLY_276
SER_278
VAL_279
Time (nsec)
≥4
3
2
1
0
# of contacts

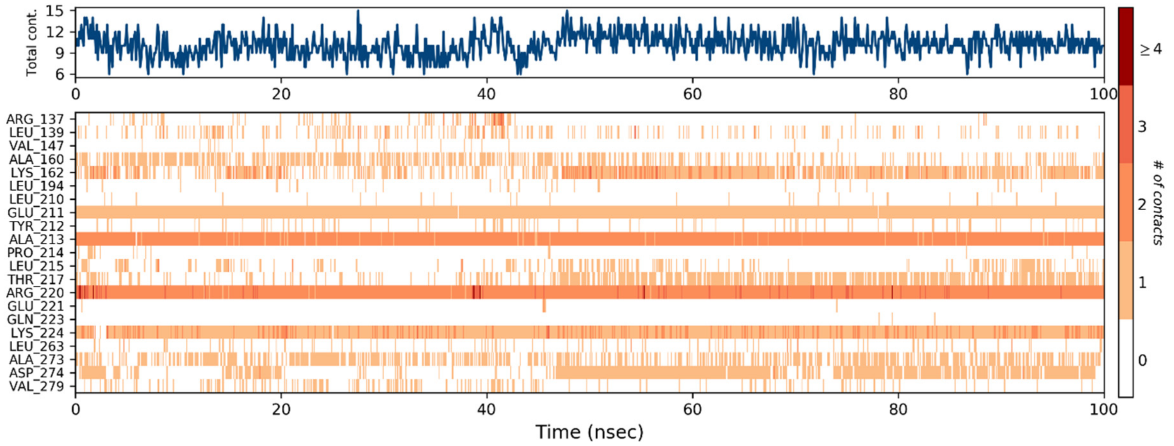
Total cont.
15
12
9
6
0
20
40
60
80
100
ARG_137
LEU_139
VAL_147
ALA_160
LYS_162
LEU_194
LEU_210
GLU_211
TYR_212
ALA_213
PRO_214
LEU_215
THR_217
ARG_220
GLU_221
GLN_223
LYS_224
LEU_263
ALA_273
ASP_274
VAL_279
Time (nsec)
≥4
3
2
1
0
# of contacts

Figure S8. The average number of interactions (total-up, per specific residue-bottom) over the simulation time for Aurka. The subsequent figures present the complexes in the order: **1** (top), **2**, **3**, AZD1152 (bottom).
